# Escalation of CTX-M-producing extensively drug-resistant *Shigella* spp. in Kolkata, India, following the COVID-19 pandemic

**DOI:** 10.1128/spectrum.02824-25

**Published:** 2026-07-24

**Authors:** Puja Bose, Gourab Halder, Pratanu Kayet, Goutam Chowdhury, Surajit Basak, Deboleena Roy, Daisuke Imamura, Shin-ichi Miyoshi, Masatomo Morita, Thandavarayan Ramamurthy, Hemanta Koley, Santasabuj Das, Asish Kumar Mukhopadhyay

**Affiliations:** 1 Division of Bacteriology, ICMR-National Institute for Research in Bacterial Infections 30170 , Kolkata, India; 2 Division of Bioinformatics, ICMR-National Institute for Research in Bacterial Infections 30170 , Kolkata, India; 3 Collaborative Research Centre of Okayama University for Infectious Diseases at ICMR-NIRBI, Kolkata, India; 4 Research Center for Intestinal Health Science, Okayama University 12997, Okayama, Japan; 5 Department of Bacteriology I, National Institute of Infectious Diseases 13511, Shinjuku City, Tokyo, Japan; 6 Division of Clinical Medicine, ICMR-National Institute for Research in Bacterial Infections 30170 , Kolkata, India; University of Saskatchewan, Saskatoon, Canada; Institut National de Santé Publique du Québec, Québec, Canada

**Keywords:** dysentery, AMR, XDR, *Shigella *spp., CTX-M, plasmid, transconjugants, PFGE, WGS

## Abstract

*Shigella* spp. is recognized by the World Health Organization as a high-priority pathogen due to its global prevalence, unique pathogenic mechanisms, and growing antimicrobial resistance (AMR). Nearly half of all *Shigella* strains worldwide are now multidrug-resistant (MDR), and the emergence of extensively drug-resistant (XDR) variants—resistant to ciprofloxacin, ceftriaxone, and azithromycin—has severely limited effective treatment options. The present study is based on prospective laboratory surveillance involving 323 *Shigella* isolates collected during 2021–2023, with pre-COVID-19 pandemic data included from a previously published study solely for historical comparison. The presence of antibiotic resistance genes (ARGs) was investigated, and whole-genome sequencing (WGS) was performed on representative isolates to assess phylogenetic relatedness with global isolates. Approximately 10% of isolates exhibited resistance to third-generation cephalosporins. While only 3% of *Shigella* isolates carried the *bla*_CTX-M-15_ gene from 2013 to 2019, its prevalence increased to 26% by 2022–2023. Among 38 ceftriaxone-resistant *S. sonnei* isolates, 33 were also resistant to azithromycin, categorizing them as XDR. These isolates showed 48% clonal similarity and high phylogenetic resemblance to the isolates reported from England. Hybrid genome assembly revealed a plasmid harboring both the *bla*_CTX-M-15_ and *mphA* ARGs. Conjugation experiments and plasmid profiling confirmed the plasmid’s transferability. We report a rising trend in third-generation cephalosporin resistance among *Shigella* spp., primarily driven by the spread of extended-spectrum β-lactamase-producing *S. flexneri* and the emergence of XDR *S. sonnei*. These findings underscore the urgent need for strengthened national AMR containment strategies and enhanced international surveillance of cephalosporin-resistant *Shigella* to mitigate this growing public health threat.

**IMPORTANCE:**

*Shigella* is a leading cause of diarrheal disease globally and has been prioritized by the World Health Organization due to its rapid acquisition of antimicrobial resistance. Our prospective surveillance in Kolkata, India, reveals a worrisome escalation of third-generation cephalosporin resistance over the past decade, primarily associated with the spread of *bla*_CTX-M-15_ and the emergence of extensively drug-resistant (XDR) *S. sonnei*. The detection of plasmids carrying both *bla*_CTX-M-15_ and *mphA*, coupled with evidence of their transferability, highlights the potential for accelerated dissemination of multidrug resistance. When compared with a global data set of international genomes, the Kolkata XDR isolates were found to cluster closely with isolates reported from England. By linking local surveillance with global genomic context, our findings provide critical insights for treatment guidelines, antimicrobial stewardship, and the design of international containment strategies aimed at curbing the rise of cephalosporin- and azithromycin-resistant *Shigella*.

## INTRODUCTION

Diarrhea is one of the common causes of death among children worldwide. Shigellosis is an infectious bacillary dysentery typically associated with the consumption of contaminated food and water. The causative agent, *Shigella,* is one of the leading causes of diarrheal mortality among all ages, accounting for 212,438 deaths (1). Although shigellosis is a global concern, its impact is disproportionately high in low- and middle-income countries, such as India, where isolation rates range from 4% to 6% across various regions, but a recent study from India has revealed that the actual prevalence of shigellosis was 18.3% in childhood diarrhea (2–5). Shigellosis is considered a highly contagious disease with a very low infectious dose (10–100 organisms) and highly transmissible in poor hygienic areas, affecting high-risk groups like children, aged individuals, immunocompromised patients, and men who have sex with men (MSM) (6). Although shigellosis is a self-limiting disease, it sometimes triggers severe and life-threatening complications depending on the infecting species. Antimicrobial therapy is useful in reducing shedding of the pathogen in the feces, reducing the duration of illness, and the risk of subsequent complications. Thus, timely administration of effective antibiotics remains a cornerstone in the management of dysenteric *Shigella* infections.

According to current guidelines, ciprofloxacin is recommended as the first-line treatment for pediatric shigellosis, followed by ceftriaxone or azithromycin as second-line options (7). However, the increasing global emergence of multidrug-resistant (MDR) *Shigella* strains has significantly compromised the effectiveness of these standard treatment regimens. Fluoroquinolone resistance, in particular, has become widespread, leading to the recommendation of ceftriaxone for managing ciprofloxacin-resistant infections (8, 9). Compounding this challenge is the growing prevalence of extended-spectrum β-lactamase (ESBL)-producing *Shigella* strains, which exhibit resistance to extended-spectrum cephalosporins, such as cefotaxime, ceftazidime, and ceftriaxone. The dissemination of ESBL genes, notably *bla*_CTX-M_ variants, poses a significant threat to therapeutic efficacy. Recent surveillance reports have indicated that approximately 5% of *Shigella* infections are caused by extensively drug-resistant (XDR) isolates, defined by resistance to all commonly recommended oral antibiotics (https://www.cdc.gov/han/2023/han00486.html). Notably, outbreaks of XDR *Shigella* among MSM communities have been reported across multiple regions worldwide, further underscoring the public health implications (10–12).

In this study, we aimed to investigate the prevalence and genetic characteristics of ESBL-producing *Shigella* isolates from diarrheal patients over a multi-year period starting from 2021. Special emphasis was placed on their association with azithromycin resistance and the underlying molecular determinants, in order to better understand the evolving antimicrobial resistance (AMR) landscape and to inform public health interventions.

## RESULTS

### Study population, antibiotic susceptibility, and detection of ARG

From 2021 to 2023, 323 (7.5%) *Shigella* spp. were isolated from 4,291 enrolled diarrheal patients admitted to the Infectious Diseases Hospital and B. C. Roy Children’s Hospital, Kolkata, India. Among the *Shigella* spp., 69.7%, 27.9%, 1.5%, and 0.9% of the isolates were identified as *S. flexneri*, *S. sonnei*, *S. dysenteriae,* and *S. boydii*, respectively. Antimicrobial susceptibility testing (AST) results showed that among 323 of the isolates, 86 (26.6%) exhibited a third-generation cephalosporin-resistant phenotype, while the PCR assay revealed that 77 (23.8%) of the isolates harbored the *bla*_CTX-M-15_ gene. Testing for the presence of other antibiotic resistance genes (ARGs) revealed that 48% (37/77) of isolates had an XDR trait, i.e., resistant to ampicillin, third-generation cephalosporin, cotrimoxazole, and azithromycin. Most of these isolates carried *bla*_CTX-M-15_ and *mphA*, *dfrA,* and *sul* genes. Isolates having only the *bla*_CTX-M-15_ gene were considered ESBL producers, while the others with both third-generation cephalosporin and macrolide resistance were denoted XDR *Shigella*. Table 1 represents the number of ESBL-producing and XDR *Shigella* spp. identified in this study.

**TABLE 1:** Serogroup distribution of ESBL-producing *Shigella* and XDR *Shigella^a^*

	Only *bla*_CTX-M_-positive (ESBL)				*bla*_CTX-M_ and *mphA*-positive (XDR)			
	SD	SF	SB	SS	SD	SF	SB	SS
2013–2019	0	8	0	4	0	0	0	0
2021–2023	1	33	1	5	1	3	0	33

^*a*^ SF, *S. flexneri*; SS, *S. sonnei;* SD, *S. dysenteriae*; SB, *S. boydii*.

**Fig 1:**
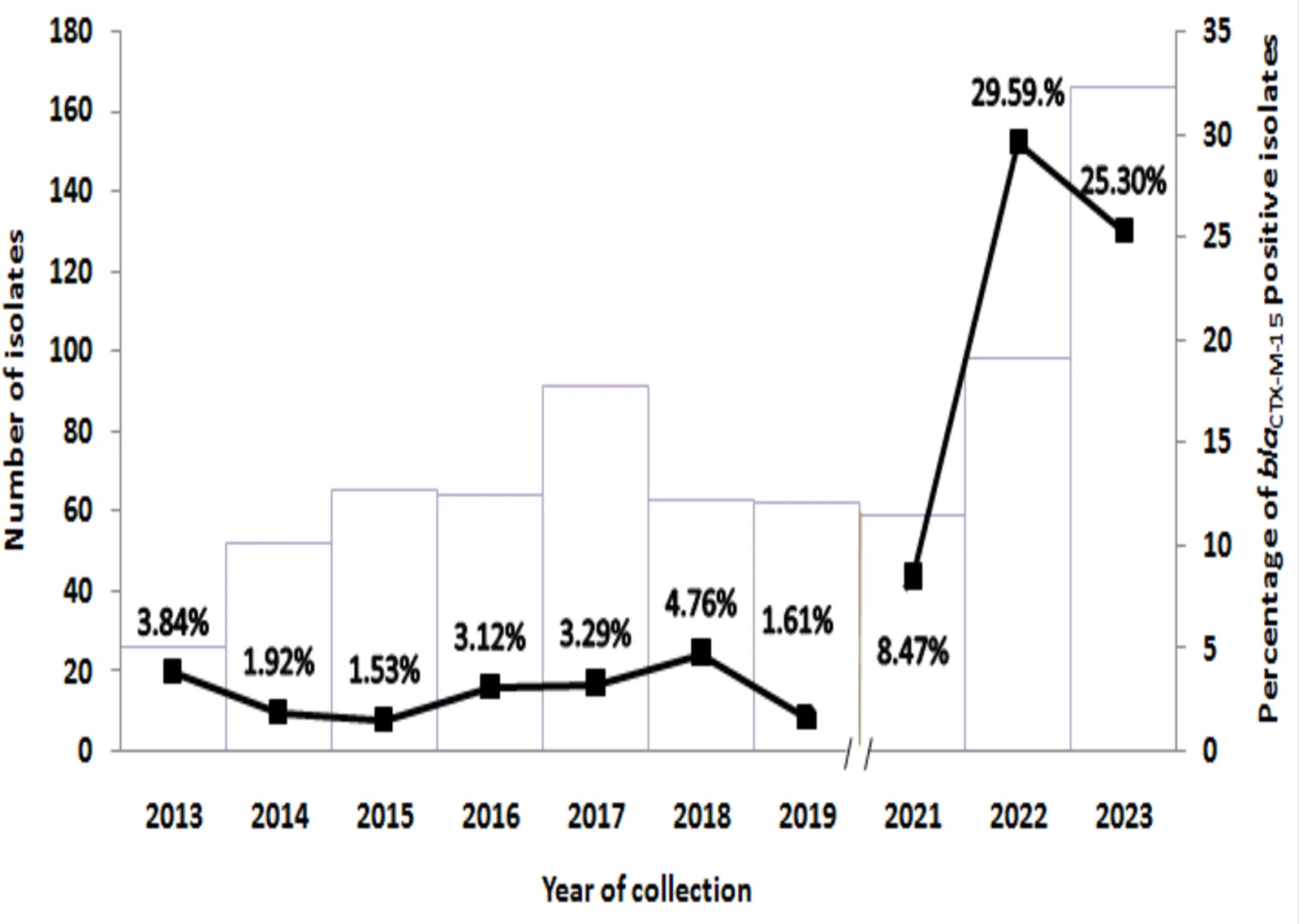
*bla*_CTX-M-15_ positivity rate among *Shigella* isolates during pre-COVID-19 (2013-2019) and post-COVID-19 (2021-2023) period.

Our previous study conducted during 2013–2019 identified 12 *Shigella* spp. isolates carrying only *bla*_CTX-M-15_, and none had the combination of *bla*_CTX-M-15_ and *mphA* (13). In addition, they were phenotypically susceptible to azithromycin. The diarrheal surveillance was discontinued in 2020 due to the COVID-19 pandemic. During the 7 years of the pre-COVID period, the *bla*_CTXM-15_-positivity rate was 2.9%. After the pandemic, the *bla*_CTX-M-15_-positivity escalated to 21.1% (Fig. 1).

**Fig 2:**
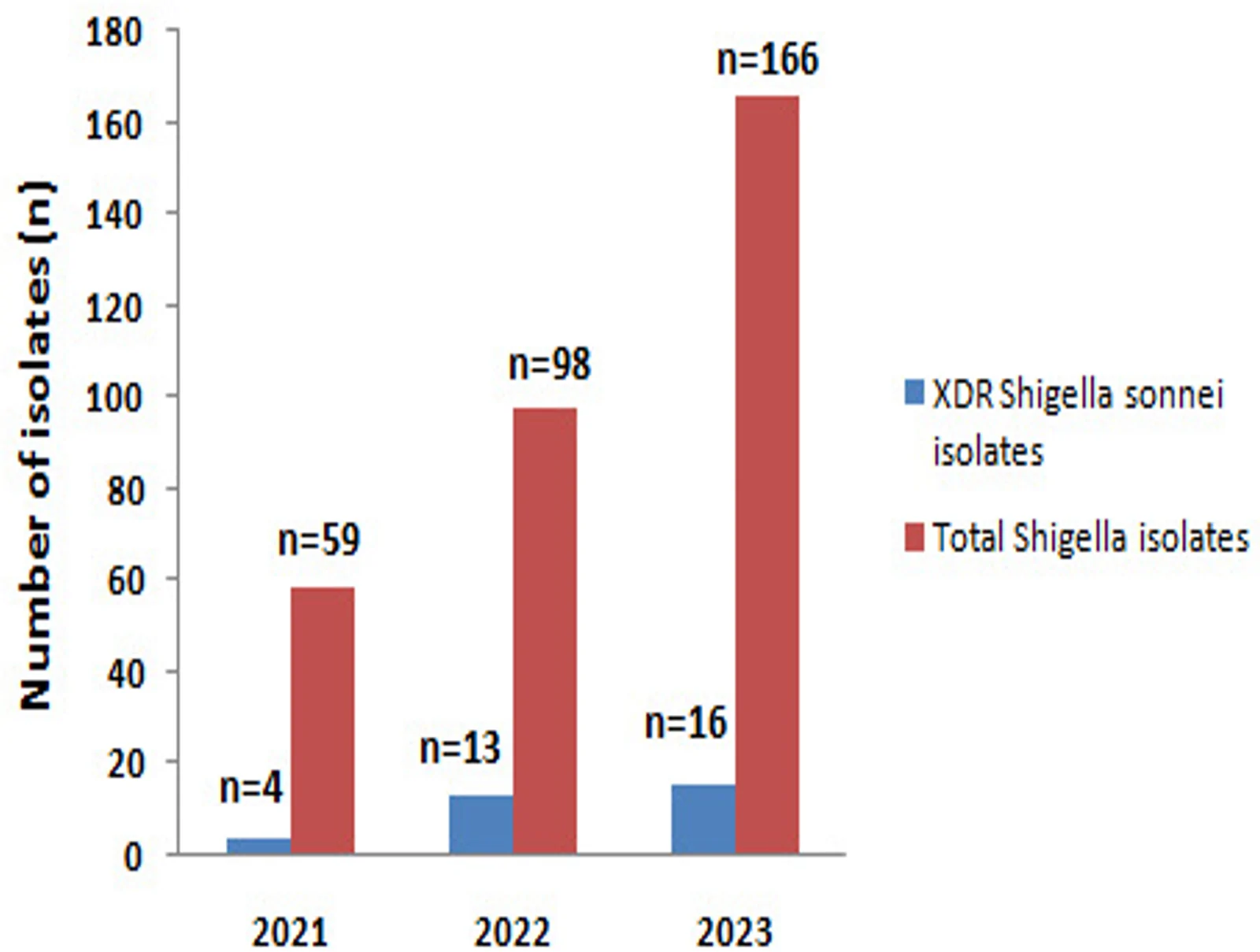
Isolation data regarding total XDR *Shigella sonnei* with respective post-pandemic years.

XDR was mainly detected among *S. sonnei* (33/37, 89.2%), as this species was resistant to ampicillin, ciprofloxacin, azithromycin, co-trimoxazole, and cephalosporins. Figure 2 represents the isolation data of XDR *S. sonnei* with respective post-COVID-19 pandemic years.

In the present study, *Shigella* isolates harbored only *bla*_CTX-M-15_. Among 40 ESBL-producers, none were identified as macrolide resistant. All the isolates are ampicillin and ceftriaxone resistant, with minimum inhibitory concentration (MIC) >256 and 64 mg/L, respectively. About 90% (36/40) isolates were also resistant to cefotaxime, with an average MIC of >64 mg/L and 40% of the isolates (16/40) had *bla*_OXA-1_. None of them carried *bla*_SHV_ and *bla*_TEM_ genes. About 52% (21/40) of the isolates showed resistance to co-trimoxazole, with MIC >16/304 mg/L; among these, 85.7% (18/21) were *dfrA*-positive and 23.8% (5/21) were *sul2*-positive.

All the tested ESBL-producing isolates remained resistant to ciprofloxacin, and 65% (26/40) carried plasmid-mediated quinolone resistance gene *qnrS* and one with *qnrB*. Apart from ARGs, integron cassettes were also detected in *Shigella* spp. The majority of them (90%, 36/40) were positive for class I integrase. Interestingly, 2 had a typical class-1 cassette, but 30 harbored an atypical class-1 cassette. However, among 33 class-2 integrase-positive isolates, only 20 had a class-2 integron cassette that included 15 *S. flexneri* (2 kb), *S. sonnei,* and *S. dysenteriae* (1.5 kb each).

All the XDR isolates were resistant to azithromycin, with MICs ranging from 32 to 96 mg/L and carried *mphA,* and only seven had *ermB*. They were resistant to ampicillin, with the MIC of 8–32 mg/L. For co-trimoxazole, the MIC range was 4/76–16/304 mg/L, with the corresponding ARGs *dfrA* (100%, 37/37), *sul2* (16.2%, 6/37), and *sul1* (100%, 37/37). Table 2 shows the AST results along with the genetic determinants of XDR *S. sonnei*.

**TABLE 2:** List of XDR *S. sonnei* ioslates with antimicrobial resistance (AMR) and ARG details^*a*^

Isolate	Ciprofloxacin	Azithromycin		Ceftriaxone		Cotrimoxazole		Ampicillin	
	ARG	MIC mg/L	ARG	MIC mg/L	ARG	MIC mg/L	ARG	MIC mg/L	ARG
B13190	*qnrS*	48	*mphA*	>64	*bla*_CTX-M-15_	40	*dfr, sul1*	>32	*bla*_OXA-1_
B 13114	*qnrS*	96	*mphA*	>64	*bla*_CTX-M-15_	160	*dfr, sul1*	>32	*bla*_OXA-1_
B 12931	*qnrS*	48	*mphA*	>64	*bla*_CTX-M-15_	160	*dfr, sul1*	32	*bla*_OXA-1_
B 12988	*qnrS*	48	*mphA*	>64	*bla*_CTX-M-15_	80	*dfr, sul1*	16	*bla*_OXA-1_
M 11	*qnrS*	96	*mphA*	>64	*bla*_CTX-M-15_	80	*dfr, sul1*	32	*bla*_OXA-1_
B 13619	*qnrB*	64	*mphA*	<1	*bla*_CTX-M-15_	>320	*dfr, sul1*	>32	*bla*_OXA-1_
B 13539	*qnrS*	48	*mphA*	32	*bla*_CTX-M-15_	160	*dfr, sul1*	16	*bla*_OXA-1_
B 13532	*qnrS*	48	*mphA*	32	*bla*_CTX-M-15_	160	*dfr, sul1*	16	*bla*_OXA-1_
M 164A	*qnrS*	64	*mphA*	>64	*bla*_CTX-M-15_	160	*dfr, sul1, sul2*	>32	*bla*_OXA-1_
B 13666	*qnrS*	32	*mphA*	>64	*bla*_CTX-M-15_	>320	*dfr, sul1, sul2*	>32	*bla*_OXA-1_
B 13500	*qnrS*	64	*mphA*	8	*bla*_CTX-M-15_	>320	*dfr, sul1*	>32	*bla*_OXA-1_
M 165	*qnrS*	64	*mphA*	>64	*bla*_CTX-M-15_	160	*dfr, sul1, sul2*	>32	*bla*_OXA-1_
M 153B	*qnrB*	>256	*mphA, ermB*	8	*bla*_DHA-1_	>320	*dfr, sul1*	16	*bla*_OXA-1_
I 120	*qnrS*	64	*mphA*	>64	*bla*_CTX-M-15_	>320	*dfr, sul1*	>32	*bla*_OXA-1_
B 13483	*qnrS*	64	*mphA*	>64	*bla*_CTX-M-15_	80	*dfr, sul1, sul2*	>32	*bla*_OXA-1_
I 377	*qnrB*	64	*mphA, ermB*	<1	*bla*_CTX-M-15_	160	*dfr, sul1*	>32	*bla*_OXA-1_
I 348	*qnrS*	64	*mphA*	8	*bla*_CTX-M-15_	160	*dfr, sul1, sul2*	>32	*bla*_OXA-1_
B 13816	*qnrS*	32	*mphA*	8	*bla*_CTX-M-15_	160	*dfr, sul2*	16	*bla*_OXA-1_
I 13546	*qnrS*	96	*mphA, ermB*	>64	*bla*_CTX-M-15_	160	*dfr, sul1*	>32	*bla*_OXA-1_
I 13436	*qnrS*	NZ	*mphA*	>64	*bla*_CTX-M-15_	160	*dfr, sul1*	>32	*bla*_OXA-1_
I 13579	*qnrS*	NZ	*mphA*	>64	*bla*_CTX-M-15_	>320	*dfr, sul1*	>32	*bla*_OXA-1_
I 13578	*qnrS*	NZ	*mphA, ermB*	>64	*bla*_CTX-M-15_	160	*dfr, sul1*	>32	*bla*_OXA-1_
I 13299	*qnrS*	NZ	*mphA*	8	*bla*_CTX-M-15_	80	*dfr, sul1*	>32	*bla*_OXA-1_
I 13668	*qnrS*	64	*mphA*	>64	*bla*_CTX-M-15_	160	*dfr, sul1*	>32	*bla*_OXA-1_
I 13437	*qnrS*	NZ	*mphA*	>64	*bla*_CTX-M-15_	40	*dfr, sul1*	>32	*bla*_OXA-1_
M 316	*qnrS*	NZ	*mphA, ermB*	4	*bla*_CTX-M-15_	160	*dfr, sul1*	>32	*bla*_OXA-1_
B 14489	*qnrS*	96	*mphA, ermB*	8	*bla*_CTX-M-15_	160	*dfr, sul1*	>32	*bla*_OXA-1_
B 14475/2	*qnrS*	48	*mphA, ermB*	2	*bla*_TEM-1B_, *bla*_CTX-M-15_	160	*dfr, sul1*	8	*bla*_OXA-1_
I 14197	*qnrS*	NZ	*mphA*	>64	*bla*_CTX-M-15_	>320	*dfr, sul1*	>32	*bla*_OXA-1_
I 14135	*qnrS*	96	*mphA*	8	*bla*_CTX-M-15_	>320	*dfr, sul1*	>32	*bla*_OXA-1_
M 348	*qnrS*	NZ	*mphA*	>64	*bla*_CTX-M-15_	160	*dfr, sul1, sul2*	>32	*bla*_OXA-1_
I 14493	*qnrS*	96	*mphA, ermB*	>64	*bla*_CTX-M-15_	>320	*dfr, sul1*	>32	*bla*_OXA-1_
GH_PEER	*qnrS*	96	*mphA*	>64	*bla*_CTX-M-15_	>320	*dfr, sul1*	>32	*bla*_OXA-1_

^*a*^ MIC breakpoints (mg/L): ciprofloxacin >1; azithromycin >32; ceftriaxone >4; cotrimoxaole >4/76; ampicillin >32.

All the *S. sonnei* isolates remained resistant to ciprofloxacin (MIC > 4 mg/L) by having plasmid-mediated quinolone resistance genes (*qnr*), 3 with *qnrB* and 30 with *qnrS*. Third-generation cephalosporin resistance was quite prominent, as the isolates were resistant to both ceftriaxone and cefotaxime with a higher range of MIC. Almost all the *S. sonnei* isolates had *bla*_CTX-M-15_ and a few had *bla*_DHA-1_ and *bla*_TEM-1B_ (Table 2). For integron gene cassette, all had class I integrase with different types of typical and atypical cassettes. Among 19 class-2 integrase positive strains, 16 *S. sonnei* had 1.5 kb cassette, and 3 *S. flexneri* had 2 kb cassette.

### Pulsed-field gel electrophoresis (PFGE)

PFGE profile analysis of representative XDR *S. sonnei* (*n* = 31) identified four clusters (Fig. S1), with five belonging to the first cluster, with 63.1% similarity, whereas another eight isolates belonged to the second cluster, and the third cluster shared 63.3% similarity among nine isolates. The fourth cluster (*n* = 7) showed 45.2% similarity to the other three clusters. Isolate GH_PEER shares 55% similarity with the first three clusters. While isolate B14475/2 revealed a distinctive banding pattern with 33.5% similarity to others.

### Whole genome sequence (WGS) and phylogenetic analyses

**Fig 3:**
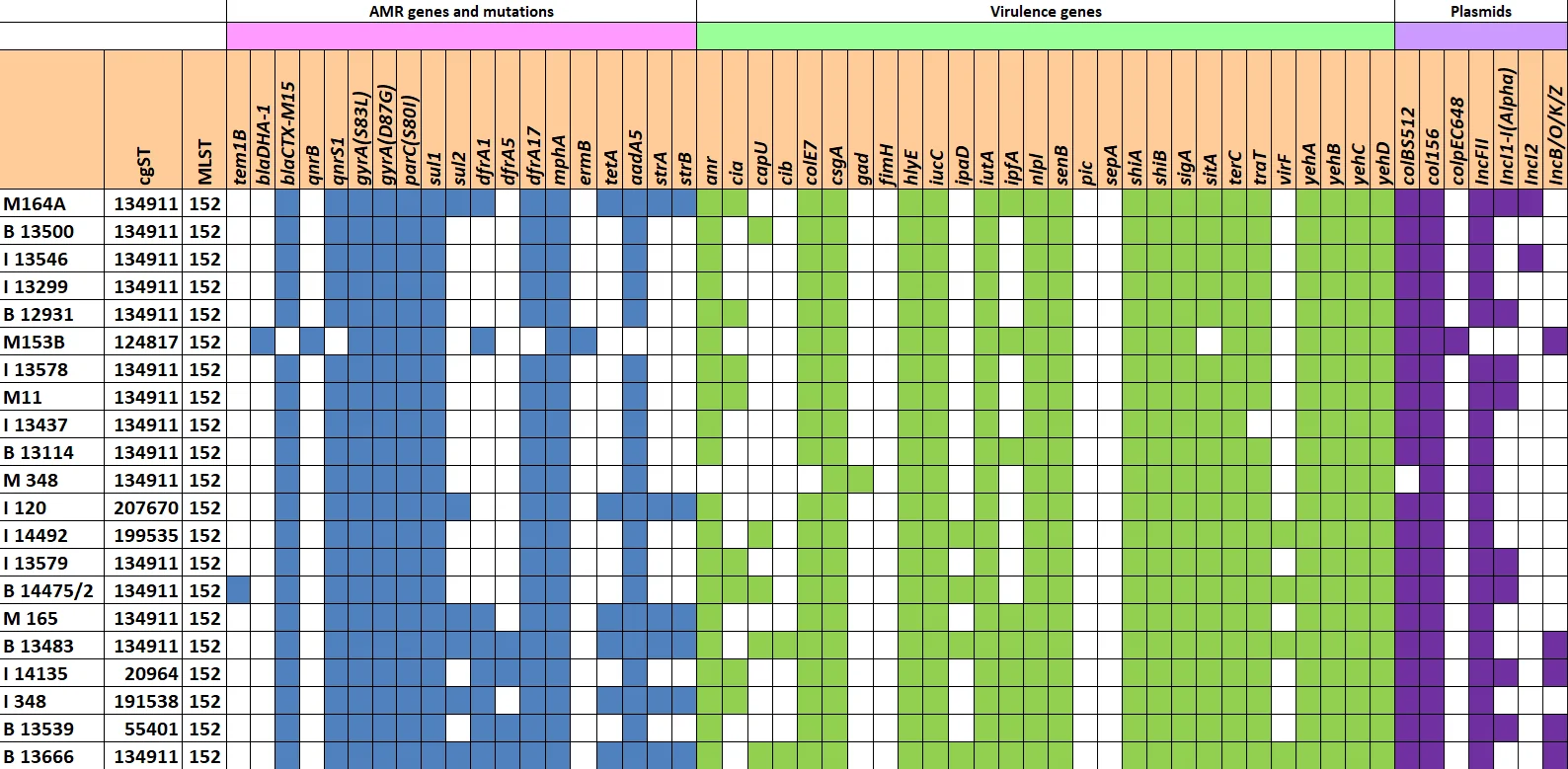
Genetic profile of representative XDR *S. sonnei* derived from WGS showing the presence of different antibiotic-resistant genes, plasmids, and virulence genes.

**Fig 4:**
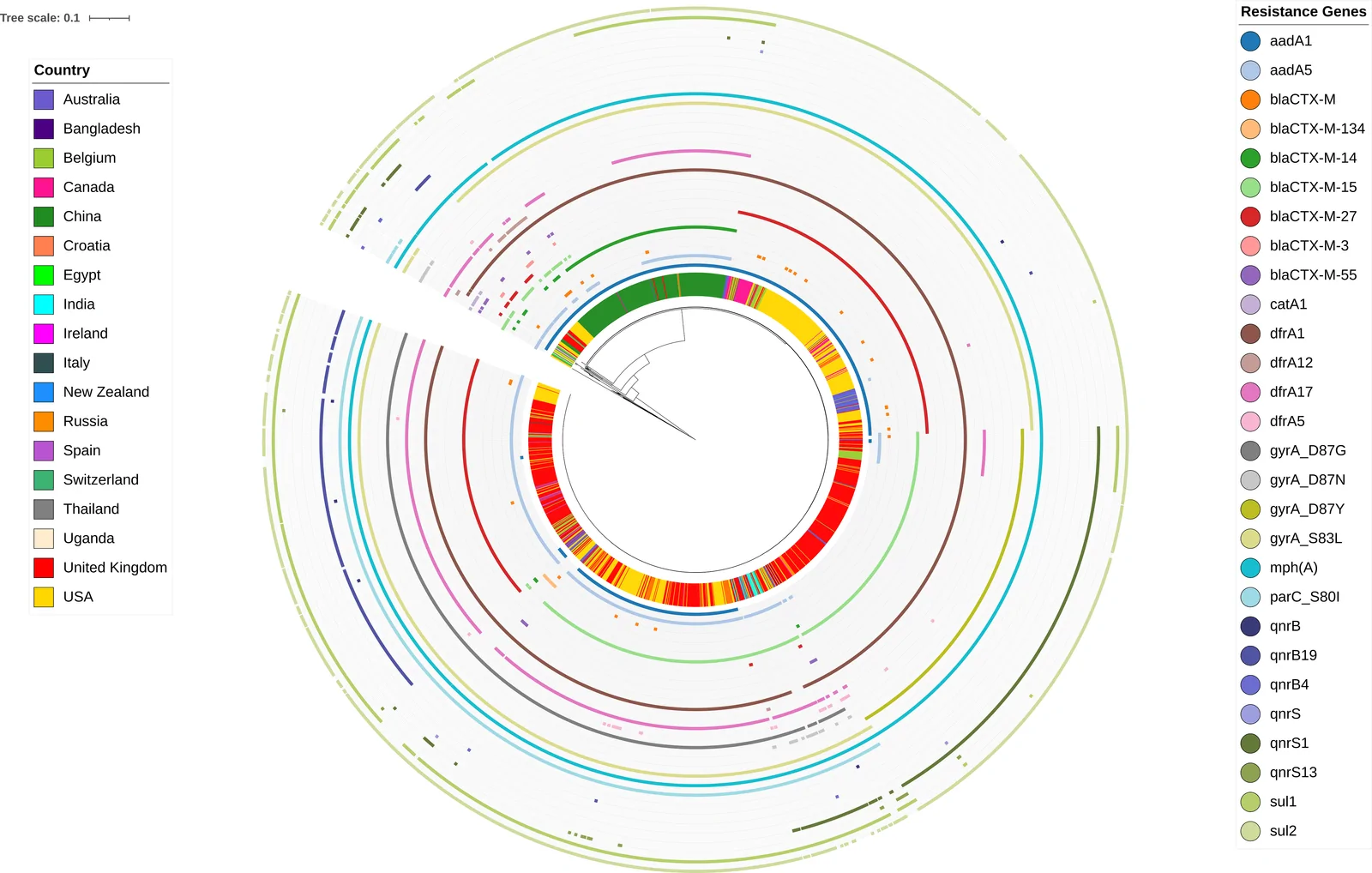
Maximum-likelihood tree of the XDR *S. sonnei* isolates collected in Kolkata from 2020 to 2023 (*n* = 21) and the selected international representative XDR *S. sonnei* isolates (*n* = 1,888). The tree was rooted with *S. sonnei* 5G.

**Fig 5:**
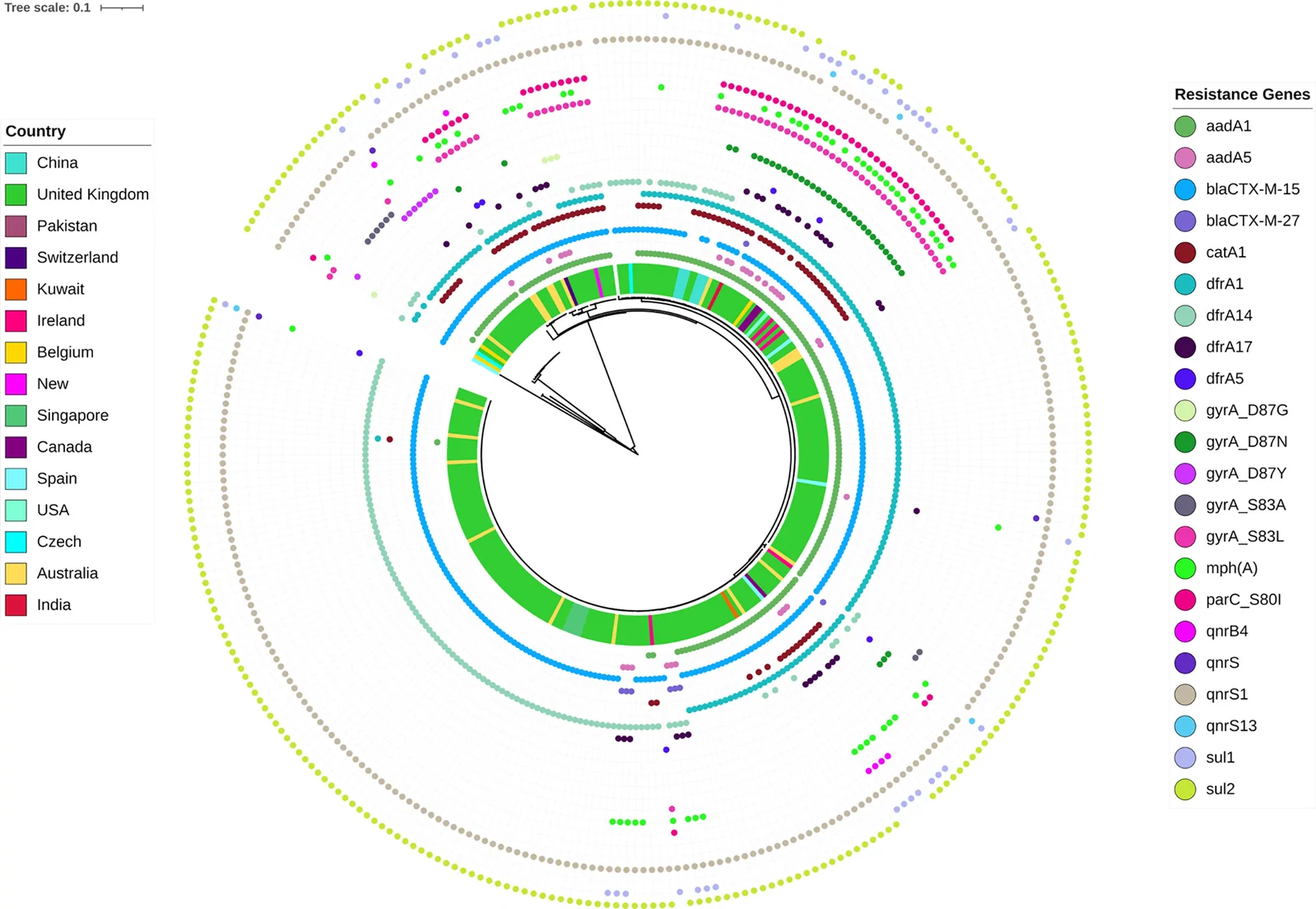
Maximum-likelihood tree of an XDR *S. flexneri* strain from Kolkata and the selected international representative XDR *S. flexneri* isolates (*n* = 297). The tree was rooted through *S. flexneri 2457T*.

All genome assemblies used in this study met the predefined quality criteria (Table S1), ensuring the robustness of the comparative genomic analyses. WGS of representative XDR *S. sonnei* revealed that all the isolates belong to ST152, the globally distributed common ST in this species. In the cgMLST, 15 isolates showed type “134911.” All the isolates harbored several ARGs. Notably, the isolate B14475/2 contained *bla*_TEM-1B_ apart from *bla*_CTX-M-15_, and the other isolate M153B had *bla*_DHA-1_ instead of *bla*_CTX-M-15_. All the isolates had triple mutations (*gyrA* S83L, *gyrA* D87G, *parC* S80I) in the quinolone resistance-determining region (QRDR). All the isolates had *qnrS1*/*qnrB* ARGs that also encode resistance to quinolone. WGS analysis identified a single isolate that had the macrolide-resistant gene *ermB*, but *mphA* was present in the rest of the isolates. Almost all the sequenced isolates carried an IncFII type plasmid, but the isolate M153B harbored an IncB/O/K/Z type plasmid. Figure 3 presents the genetic profile consisting of different ARGs, plasmids, and virulence-encoding genes. WGS analysis of two different *Shigella* serogroups is presented in Table 3. Phylogenetic analysis of Kolkata XDR *S. sonnei* and *S. flexneri* revealed high similarity with the isolates reported from the United Kingdom (Fig. 4 and 5).

**TABLE 3:** WGS details of representative strains from other *Shigella* groups

Strain	*Shigella* spp.	ARGs	Plasmid type	MLST type	cgMLST
B 13618	*S. flexneri 2a*	*qnrS1, bla*_CTX-M-15_ *, bla*_OXA1_ *, sul1, dfrA1, mphA, catA1, aadA1, aadA5, dfrA17*	IncFII	ST245	64133
I 182	*S. dysenteriae*	*aadA, strA, strB, bla*_CTXM-15_ *, mphA, qnrS1, sul1, sul2, tetB, dfrA, dfrA17*	IncFII	ST148	45653

### Nanopore sequencing and hybrid assembly

**Fig 6:**
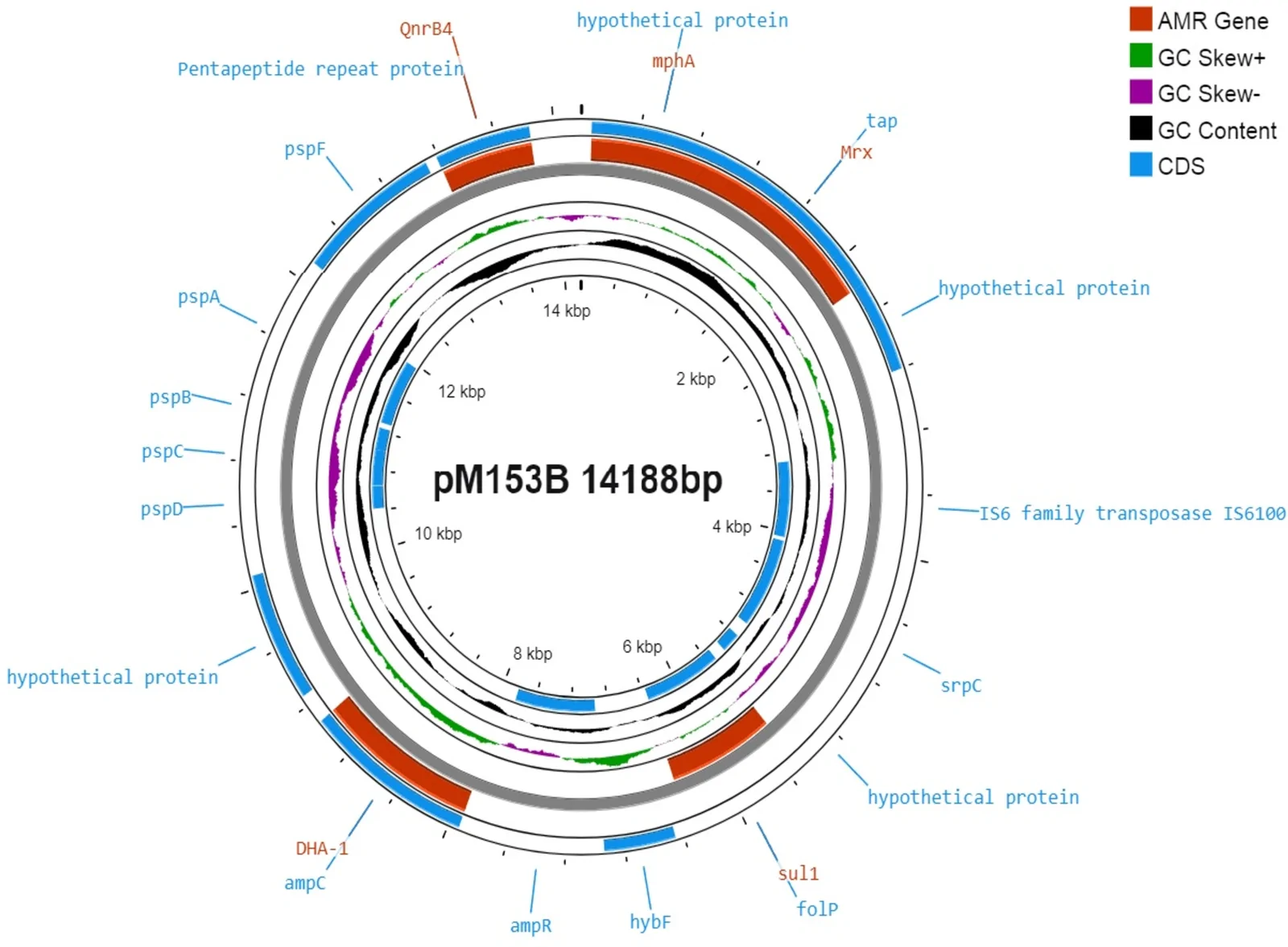
Hybrid assembly illustrated that *mphA* and *bla*_DHA_ are in the plasmid backbone (M153B).

The long-read and hybrid assembly of a *S. sonnei* isolate M153B showed that the third-generation cephalosporin resistance-encoding gene (*bla*_DHA-1_) and azithromycin-resistant gene *mphA* were present in a transferable plasmid (Fig. 6), and this observation was supported by the conjugation assay.

### Conjugation and transfer of resistant plasmid

*Escherichia coli* J53 transconjugants were resistant to erythromycin, ampicillin, and azithromycin, similar to the donor *S. sonnei* (M153B and M164A), *S. dysenteriae* (I-182), and *S. flexneri* (B-13618) (Table 4). The transconjugants showed an MIC of azithromycin of ≥256 mg/L. The *mph*A gene was confirmed in the transconjugant’s plasmid DNA by PCR. The plasmid profile of the recipient and transconjugant is shown in Fig. S2. Although the WGS data of IDH 12931 showed the presence of *bla*_CTX-M-15_, the conjugation experiment failed to produce any transconjugant. We hypothesize that *bla*_CTX-M-15_ may be located in the chromosome. Furthermore, hybrid assembly by Nanopore sequencing validated this view (Fig. S3).

**TABLE 4:** Phenotypic and plasmid characteristics of *S. sonnei* and transconjugants

Isolate	Donor isolate		Transconjugant		Conjugation efficiency
	Resistance profile	ARGs	Resistance profile	ARGs	
*S. sonnei* (M153B)	AZM, E, AM, CRO, SXT	*mphA*, *bla*_DHA-1_, *qnrB*	AZM, E, AM, AZD	*mphA*, *bla*_DHA-1_, *qnrB*	6.6 × 10^−6^
*S. sonnei* (M164A)	AZM, E, AM, CRO, SXT	*mphA*, *bla*_CTX-M-15_, *qnrS*	AZM, E, AM, CTX, CRO, AZD	*mphA*, *bla*_CTX-M-15_, *qnrS*	2.4 × 10^−6^
*S. sonnei* (I 12931)	AZM, E, AM, CRO, SXT	*mphA*, *bla*_CTX-M-15_, *qnrS*	–^*a*^	–	–
*S. flexneri* (B 13618)	AZM, E, AM, CRO, SXT	*mphA*, *bla*_CTX-M-15_, *qnrS*	AZM, E, AM, CTX, CRO, SXT, AZD	*mphA*, *bla*_CTX-M-15_, *qnrS*	5.3 × 10^−6^
*S. dysenteriae* (I 182)	AZM, E, AM, CRO, SXT	*mphA*, *bla*_CTX-M-15_, *qnrS*	AZM, E, AM, CTX, CRO, SXT, AZD	*mphA*, *bla*_CTX-M-15_, *qnrS*	3.2 × 10^−6^

^*a*^ “–” indicates absence of resistance profile, ARG, and conjugation efficiency data, as strain I12931 failed to produce any transconjugants.

### Plasmid stability analysis

The stability of the plasmids in *E. coli* transconjugants was evaluated through serial passaging experiments conducted over 5 days in antibiotic-free LB broth. The isolate harboring the plasmid demonstrated a high level of plasmid stability. After 6 days of continuous passaging, 100% of the examined colonies retained the plasmid, as confirmed by PCR screening for the associated ARGs. These findings indicate that the plasmid remains highly stable within the host isolate under the experimental conditions, with no plasmid loss throughout the study period.

## DISCUSSION

India is one of the largest producers and consumers of antimicrobials globally; hence, the task of curbing AMR is a huge challenge. The emergence of ESBL *Shigella* and the appearance of XDR *Shigella* in Kolkata are major public health concerns. In our previous observation, only a limited number (*n* = 12) of third-generation resistant *Shigella* isolates have been reported in Kolkata (13). None of the previous isolates, including *S. sonnei* (4/12) and *S. flexneri* (8/12), were resistant to the macrolide class of antibiotics but remained resistant to other antimicrobials like fluoroquinolones, ampicillin, and co-trimoxazole. In this study, we observed that the emerging trend of ESBL-producing *S. flexneri* has increased in distinct serotypes. Another noteworthy trend is the emergence of XDR *S. sonnei* strains resistant to azithromycin, ampicillin, cotrimoxazole, and fluoroquinolones. To the best of our knowledge, there have been no such reports of XDR *Shigella* spp. in India.

During the COVID-19 epidemic, India’s socio-economic landscape was marked by increased vulnerability across multiple dimensions. Poverty surged, with an estimated 19% of urban non-poor becoming poor and over 60% of rural households vulnerable (14). Financial distress intensified, particularly in districts with high infection rates (15). The crisis disproportionately affected informal workers and migrants (15, 16). Districts with economically deprived populations experienced higher mortality (16). However, government food subsidies and migrant remittances helped mitigate from adverse effects. In addition, the COVID-19 pandemic period of 2020–2021 triggered a paradoxical and concerning shift in antibiotic prescribing patterns, characterized by a surge in usage despite a viral etiology. Contrary to expectations of reduced antibiotic use due to lockdowns, the first wave led to a significant increase in consumption. Meta-analyses and observational studies have demonstrated that in multiple settings, 70%–90% of hospitalized COVID-19 patients received empiric antibiotics, including ceftriaxone and azithromycin, with usage patterns poorly aligned with actual bacterial disease burden and stewardship principles (17–21). An interrupted time series analysis of private sector sales estimated that the COVID-19 epidemic contributed to an excess of 216 million doses of antibiotics between June and September 2020, with azithromycin alone accounting for 38 million excess doses, likely driven by its repurposing for COVID-19 management despite a low incidence of bacterial co-infection (22). This pattern underscores a critical disjunction between healthcare infrastructure expansion and the quality of care delivered, as the empirical use of broad-spectrum antibiotics, a pre-existing challenge highlighted by pre-pandemic studies showing 44.8% of prescriptions were empirical (23). Besides the empirical use of antimicrobials in clinical settings, another important factor leading to increased antimicrobial consumption is self-medication. The knowledge that antimicrobials recommended for COVID-19 are readily available without a prescription further facilitates the practice of self-medication. Such dynamics, compounded by limited antimicrobial stewardship during the pandemic and the high prevalence of over-the-counter antibiotic use in India, may have accelerated the expansion and clinical dominance of XDR *Shigella* clones in the post-pandemic period (24).

The prevalence of XDR *Shigella* has been reported in several countries. Following the increase in fluoroquinolone resistance, the spread of XDR *S. sonnei* has been reported for the first time in Vietnam since 2014 (25). Following this finding, the appearance of XDR *S. sonnei* has been reported in other countries, including the USA, the United Kingdom, Australia, Belgium, Spain, France, and China (10, 12, 26–31). Most of these globally reported XDR isolates were associated with MSM. In India, the MSM population is estimated to be approximately 352,000, with 4.3% of those infected with HIV (32–34). In contrast to global trends, our findings on the identification of XDR *Shigella* spp. primarily involve pediatric diarrheal cases from Kolkata, with only four XDR *Shigella* isolates identified in adult male patients. An important and concerning observation in this study is that the majority of XDR *Shigella* isolates were recovered from pediatric patients, whereas globally, the emergence and dissemination of XDR *Shigella*, particularly *S. sonnei*, has been predominantly associated with transmission within MSM networks. This divergence from the global epidemiological pattern suggests that these high-risk resistant lineages are no longer confined to specific adult transmission networks but may have become established in the community and are now circulating widely among children.

A recent 10-year retrospective study from Israel reported a dramatic rise in ceftriaxone resistance in pediatric *S. sonnei* isolates, increasing from 3.2% in 2019 to 78% in 2021, indirectly reflecting the rapid expansion of XDR *S. sonnei* lineages in the region (35). Although comprehensive AMR profiling was not performed in that study, the marked increase in third-generation cephalosporin resistance strongly supports the notion of accelerated acquisition of resistance determinants among *S. sonnei* circulating in the pediatric population, supporting our findings.

These findings indicate a worrying shift in the epidemiology of XDR *Shigella*, where highly resistant isolates traditionally linked to specific adult transmission routes are now posing a direct therapeutic challenge in children, a population with limited treatment options. This transition represents a significant public health concern and underscores the urgent need for strengthened pediatric surveillance and antimicrobial stewardship. This difference in demographic distribution underscores the potential for varied transmission dynamics in the Indian context and highlights the urgent need for enhanced surveillance, antimicrobial stewardship, and targeted public health interventions.

Several studies from different countries have revealed that ST152 is the most frequent lineage among extended-spectrum cephalosporin-resistant *S. sonnei* carrying different alleles of *bla*_CTX-M_, some of them are co-resistant to quinolones and macrolides, like our study isolates (36–39). During COVID-19, MDR *S. flexneri* serotype 2a with the ST245 was identified as a causative agent for an outbreak in Vancouver (40). Comparison of WGS-based phylogenetic analysis of the XDR *S. sonnei* with global isolates revealed a high similarity with the UK isolates. Given that shigellosis is widely recognized as a travel-associated disease, international travelers may play a pivotal role in introducing and disseminating resistant strains within local populations.

Increase in azithromycin resistance in *S. sonnei* has been previously reported (41). This drug has been widely used in COVID-19 patients, and this could be a plausible reason for the emergence of macrolide-resistant ESBL *S. sonnei*. Prior investigations have often implicated *bla*_CTX-M-27_ in conferring third-generation cephalosporin resistance in *S. sonnei* (30, 41). In our study, the majority of the *S. sonnei* strains carried *bla*_CTX-M-15_ reports from other countries that substantiate the current observation (12, 42, 43).

Hybrid assembly of two representative isolates in our study revealed co-localization of *bla*_DHA-1_ and *mphA* on a single 14 kb plasmid, while another isolate carrying *bla*_CTX-M-15_ lacked plasmid-mediated transfer, indicating chromosomal integration of the resistance determinant. Recently, a novel XDR *S. sonnei* carrying an AmpC-type *bla*_DHA-1_ gene was reported from Los Angeles (44).

Previous studies have documented plasmid-to-chromosome gene transfer events as an important mechanism for stabilizing antimicrobial resistance (45). Although plasmids harboring both *mphA* and *ermB* have been reported from India, these plasmids did not carry determinants for third-generation cephalosporin resistance (45). Recently, an IncFII-type plasmid co-carrying macrolide and third-generation cephalosporin resistance genes was reported in *Shigella* spp. from Bangladesh (46). Acquisition of such plasmids likely contributes to the emergence and persistence of XDR *S. sonnei* by enriching the ARG pool within the gut microbiota. Ceftriaxone resistance in *S. flexneri* was very low between 2011 and 2019, but this trend also changed during the post-COVID-19 period. Similar trends were observed in *S. flexneri* XDR strains from different countries (11, 47).

Plasmids carrying *bla*_CTX-M-15_ and *mphA* are typically associated with highly stable, conjugative IncF/IncI-type backbones that are well adapted to *Enterobacteriaceae* hosts and impose relatively low fitness costs, facilitating their persistence even in the absence of continuous antibiotic pressure. However, our long-read sequencing data suggest that in a single isolate, these resistance determinants are no longer exclusively plasmid-borne but appear to be integrated into the chromosome. This transition has important clinical and evolutionary implications.

Chromosomal integration of ESBL and macrolide resistance genes effectively decouples resistance maintenance from plasmid carriage, eliminating the metabolic burden associated with plasmid replication and reducing the likelihood of resistance loss when antibiotic pressure is withdrawn. As a result, these XDR *Shigella* lineages may achieve stable vertical inheritance of resistance traits, allowing them to persist in the population even if antimicrobial stewardship improves and cephalosporin or macrolide usage declines. In such scenarios, resistance becomes a fixed genomic trait rather than a horizontally transferable accessory element.

Clinically, this implies that reductions in antibiotic consumption alone may be insufficient to reverse resistance trends once these genes have been chromosomally assimilated. Instead, these isolates may continue to circulate as highly fit, resistant clones within communities, particularly in high-transmission settings. Furthermore, while chromosomal integration limits the immediate horizontal transfer potential compared with plasmid-borne genes, the associated mobile elements retain the capacity to mobilize these resistance determinants back onto plasmids, maintaining the possibility of future dissemination.

Reallocation of healthcare resources toward the COVID-19 response likely reduced capacity for timely diagnosis and culture-guided therapy, increasing reliance on empiric broad-spectrum antibiotics, which can promote selection of resistant organisms. Recent reports have documented increased community antibiotic consumption and delayed surveillance activities in multiple Indian states during the pandemic period, creating conditions conducive to the emergence and dissemination of resistant enteric pathogens (22, 48, 49). Taken together, disruptions in healthcare delivery, changing antibiotic use practices, and persistent socio-economic challenges in Kolkata post-COVID-19 create an environment that plausibly supports the observed escalation of CTX-M-producing XDR *Shigella* spp.

In summary, the increasing prevalence of third-generation cephalosporin-resistant *Shigella* and the emergence of XDR *S. sonnei* in India pose a serious threat to current therapeutic strategies. This underscores the urgent need to expand the surveillance of MDR *Shigella* using an integrated approach that combines rapid pathogen identification, WGS-based characterization, and detailed mapping of transmission dynamics of antimicrobial resistance (AMR) at both national and global levels.

### Limitations

We acknowledge several methodological and surveillance-related limitations that may influence the interpretation of our findings. First, the sampling framework may have introduced selection bias. The study did not include asymptomatic community carriers or non-diarrheal controls for the assessment of resistance gene carriage. Asymptomatic individuals may serve as a silent reservoir for ARG dissemination; therefore, the absence of community-based controls limits our ability to generalize these findings to the broader population for asymptomatic carriage dynamics.

Second, the surveillance structure may have resulted in an underrepresentation of subclinical infections and cases from remote or rural settings. Only patients admitted to the Infectious Diseases Hospital and Dr. B. C. Roy Hospital, including referred cases from peripheral regions, were included. Consequently, the data set predominantly reflects isolates circulating in urban Kolkata and referred patients and may not fully capture the genetic diversity and transmission dynamics of *Shigella* isolates in rural communities.

Third, exposure-related variables, including prior antibiotic use, were based on self-reported data. Such information may have affected the accuracy of exposure classification and limited the strength of associated epidemiological inferences.

Fourth, this study provides a regional genomic overview and does not incorporate comparative genomic analyses with contemporaneous isolates from other parts of the Indian subcontinent. The absence of inter-regional genomic comparison restricts assessment of broader clonality patterns and limits inference regarding large-scale dissemination of isolates across India.

In addition to these epidemiological constraints, several genomic analytical limitations should be considered. The prevalence and structural configuration of *bla*_DHA_ and *mphA* co-location were not systematically resolved across the entire XDR collection. Comprehensive locus-level structural validation was not performed for all isolates, and most genomes were derived from short-read assemblies without systematic read-mapping confirmation or alternative assembly reconciliation. Given the abundance of insertion sequences and transposase-rich regions flanking resistance determinants, assembly artifacts, such as collapsed repeats or chimeric joins, cannot be fully excluded.

The publicly available NCBI genome assemblies included in the single-nucleotide polymorphism (SNP)-based phylogenetic analysis were generated using diverse sequencing technologies and assembly pipelines, which may introduce variability in assembly quality, SNP identification, and inferred tree topology.

Plasmid typing for the majority of isolates was inferred from short-read assemblies, which limits definitive assignment of ARGs to specific plasmid backbones and may not resolve multi-replicon or mosaic plasmid architectures. Hybrid assemblies were completed for two representative isolates and demonstrated both plasmid-borne and chromosomal integration of resistance determinants; however, the limited number of long-read-resolved genomes constrains generalizability regarding structural diversity.

Finally, we did not compute pairwise SNP distances, core-genome SNP thresholds, or patristic distances, nor did we apply standardized genomic clustering criteria. Therefore, the designation of “high similarity” is based on phylogenetic topology and shared genomic features rather than quantitative distance metrics, limiting resolution for formal transmission or outbreak delineation.

Collectively, these constraints primarily affect fine-scale transmission inference, structural resolution of resistance loci, and broader epidemiological generalizability. Accordingly, conclusions regarding genomic architecture, plasmid attribution, and population-level dissemination should be interpreted within these methodological boundaries.

## MATERIALS AND METHODS

### Study population and bacterial isolates

The study included 323 *Shigella* isolates from 4,291 diarrheal patients admitted to the Infectious Diseases Hospital and B. C. Roy Children’s Hospital in Kolkata, India, between January 2021 and December 2023. Stool specimens collected from these patients were tested for common enteric infections within 2 h using standard microbiological procedures (50). Institutional ethical clearance was obtained to conduct this investigation. For the isolation of *Shigella* spp., stool samples were directly inoculated on MacConkey’s agar, Hektoen Enteric Agar, and Xylose Lysine Deoxycholate agar (BD BBL). The plates were incubated for 18–24 h at 37°C. Suspected colonies of *Shigella* were identified biochemically and further confirmed serologically by slide agglutination using commercially available antisera (Denka Seiken). *Shigella* isolates collected between 2021 and 2023 were analyzed as part of this prospective laboratory surveillance program. In addition, the previously published 2013–2019 pre-pandemic COVID-19 data set was used exclusively in Fig. 1 and Table 1 for historical comparison and was not included in any additional analyses of the 2021–2023 prospective surveillance isolates.

### AST and MIC determination

AST of currently recommended drugs for shigellosis was performed with all *Shigella* isolates using the standard Kirby-Bauer disc-diffusion method (51). Commercially available discs (BD) of ampicillin (10 µg), ciprofloxacin (5 µg), ceftriaxone (30 µg), cefotaxime (30 µg), ceftazidime (30 µg), azithromycin (15 µg), and cotrimoxazole (1.25/23.75 µg) were used in the AST. The MICs of ampicillin, ciprofloxacin, ceftriaxone, cefotaxime, and ceftazidime (Sigma Aldrich, USA) were determined using an agar dilution method. *E. coli* ATCC 25922 served as the quality control strain. The MIC for azithromycin and cotrimoxazole was determined using Etest (Biomeriux). An increase of 5 mm in the inhibitory zone around the ceftazidime/cefotaxime discs with clavulanic acid compared to ceftazidime/cefotaxime alone was detected.

### Genotypic detection of antibiotic resistance genes

Total DNA was extracted from *Shigella* strains using a DNA extraction kit (Qiagen) and tested for the presence of ARGs, such as β-lactamases (*bla*_OXA_ and *bla*_TEM_), the extended-spectrum cephalosporin resistance gene (*bla*_CTX-M_), AmpC β-lactamases (*bla*_DHA_), plasmid-mediated quinolone resistance genes (*qnrB* and *qnrS*), and sulfonamide resistance genes (*dfrA*, *sul1*, and *sul2*), following previously established PCR protocols (13). Additionally, the PCR analysis included the *mphA* and *ermB* genes (52).

### Pulsed-field gel electrophoresis

PFGE was performed following the methods of PulseNet (https://www.pulsenetinternational.org/assets/PulseNet/uploads/pfge/PNL05_Ec-Sal-ShigPFGEprotocol.pdf) to eliminate selection of the clonal isolates for the WGS process. *Xba*I restriction digestion enzyme (50 U/plug) was used to digest bacterial DNA, and PFGE was conducted in a CHEF-DRIII electrophoresis system (Bio-Rad Life Science Group, Hercules, CA, USA) for 21 h at 6 V/cm potential with switch times ranging from 5 to 35 s at 14°C. *Xba*I-digested *Salmonella enterica* serovar Brandrup H9812 was used as a molecular weight ladder. The dendrogram was constructed as previously described using FP-Quest Software, v4.5 (BioRad Laboratories, Hercules, CA, USA), applying the Dice coefficient with a band position tolerance of 1.5% (13).

### WGS analysis

Twenty-one representative XDR *S. sonnei* isolates were chosen from different PFGE clusters and sub-clusters to confirm the genetic diversity present in the collection. In addition to PFGE diversity, epidemiological and phenotypic characteristics, including patient demographic information and antimicrobial resistance profiles, were carefully considered in order to include isolates representing different resistance patterns and temporal distribution. Bacterial DNA was extracted using the DNA mini kit (Qiagen) and quantified using the Qubit 3.0 (Thermo Fisher Scientific, USA) and Nanodrop (Thermo Fisher Scientific, USA). DNA library preparation was made with the Nextera XT kit. The Illumina Novaseq 6000 platform facilitated paired-end sequencing according to the manufacturer’s v1.5 chemical procedure, featuring a read length of 2 × 150 bp and a sequencing cycle of 2 × 501 (Illumina Inc., San Diego, CA). Additionally, in order to understand the genetic background of the *mphA* and third-generation cephalosporin ARGs, long-read sequences of two representative strains were made using the SQK-LSK114 ligation sequencing kit and Nanopore MinION (Oxford, UK). Both short and lengthy raw reads, as well as de novo hybrid assembly, have been performed using Unicycler (https://github.com/rrwick/Unicycler) (53). The quality of draft genome assemblies generated from short-read sequencing data was evaluated using standard assembly metrics to ensure reliability for downstream comparative genomic and phylogenetic analyses. For each isolate, sequencing depth (coverage), total assembly length, N50, GC content, and number of contigs (>500 bp) were calculated using QUAST v5.0.2 (http://quast.sourceforge.net). Assemblies with an average coverage of ≥30×, N50 > 20 kb, and fewer than 300 contigs were considered of acceptable quality for further analysis. PILON v1.23 (http://github.com/broadinstitute/pilon/releases/) was used to rectify all erroneous mappings in the contigs generated during contig assembly. The RAST server, available at http://rast.nmpdr.org, enabled genome annotation. Artemis, a genome viewer, was employed to visualize the .gbk file. Genomic images were generated by the SNAPGene software version 4.3.8. pubMLST (https://pubmlst.org/species-id), and the ANI calculator facilitated the speciation of *Shigella* spp. The online tools ResFinder 4.7.2 (https://genepi.dk/resfinder) and PlasmidFinder 3.0.3 (https://genepi.dk/plasmidfinder) identified the ARGs and plasmid incompatibility types.

### Phylogenetic analysis

We searched the NCBI database (www.ncbi.nlm.nih.gov) for *S. flexneri* genomes satisfying the following criteria: (i) presence of the *bla*_CTX-M_*, sul,* and *dfr* gene variants, (ii) mutation in the QRDR, or *qnr* genes. As a result, 297 *S. flexneri* genomes were retrieved. Similarly, we also searched the NCBI database for *S. sonnei* genomes containing *mphA* and/or *ermB*, as well as the presence of the *bla*_CTX-M_ gene variant. In this search, we retrieved 1,888 *S. sonnei* genomes. Therefore, 1,888 *S. sonnei* (Table S2) and 297 *S. flexneri* (Table S2) downloaded genomes were used in the phylogenetic analysis.

Assembled whole-genome sequences of 1,888 *S. sonnei* and 297 *S. flexneri* were retrieved from NCBI using an in-house developed customized Python script. SNPs were identified using Snippy (v4.3.6) (github.com/tseemann/snippy), where downloaded assembled genomes are aligned to a reference genome, and SNPs were extracted from the resulting whole-genome alignment. *S. flexneri* 2457T (GenBank: AE014073) and S. sonnei strain 53G (GenBank: NC_016822) were used as the reference genomes (12, 30). Snippy maintains default parameters as alignment identity of 70% and a minimum contig size of 500 bp to ensure that only reliable, high-confidence variant calls are retained for downstream analysis.

To account for the confounding effects of horizontal gene transfer and recombination, which can introduce false SNP signals and distort phylogenetic inference, recombination-affected genomic regions were identified and masked using Gubbins (v2.3.4) (54). Gubbins operates iteratively by building successive phylogenies and identifying regions of elevated SNP density that are indicative of recombination. By default, Gubbins maintains a maximum of five iterations and a minimum of three SNPs per recombination block as the detection threshold. The resulting alignment of non-recombinant SNPs was then used to construct a maximum-likelihood phylogenetic tree using IQ-TREE (v1.6.10) (55). IQ-TREE evaluates the best-fit nucleotide substitution model using its built-in ModelFinder algorithm before tree construction. ModelFinder is an algorithm built into IQ-TREE that tests nucleotide substitution models to determine which one best describes the evolutionary patterns in our sequence data (56). The substitution model is a core mathematical assumption that underlies every step of phylogenetic tree inference. IQ-TREE uses maximum likelihood calculation, which is dependent on the substitution model used while generating the phylogenetic tree. If the wrong model is applied, the likelihood scores become inaccurate, which can lead to incorrect branch length estimates, poorly supported nodes, and even a wrong tree topology. Running ModelFinder first ensures that the tree is built on a correct substitution model. ModelFinder selects the best model by comparing candidate models using information criteria, typically the Bayesian Information Criterion. Each model is scored based on how well it fits the data while penalizing unnecessary complexity (to avoid overfitting). GTR+G came out to be the best model for phylogenetic tree building. The General Time Reversible (GTR) model is the flexible standard nucleotide model, allowing each of the six possible nucleotide substitution rates to vary independently, along with unequal base frequencies. The +G component adds a gamma distribution to model rate variation across sites, acknowledging that some positions in an alignment evolve faster than others. IQ-TREE with the GTR+G substitution, with 1,000 bootstrap replicates, to assess branch support and node reliability. The final phylogenetic tree was annotated with relevant metadata (e.g., Country, AMR gene presence [Fig. 4 and 5]), using the Interactive Tree of Life web server (http://itol.embl.de). A complete workflow diagram for phylogenetic analysis is also presented as a supplementary figure (Fig. S4). The complete command-line workflow and key scripts used for WGS processing and phylogenetic analysis are provided in File S1 to support full reproducibility of the study.

### Conjugation and transfer of resistant plasmid

Two XDR *S. sonnei* (M153B and M164A), one XDR *S. flexneri* (B13618), and one XDR *S. dysenteriae* (I182) were used as donor strains, while *E. coli* J53 (Azide^R^) was the recipient for conjugation experiments. Both the *Shigella* strains had high MICs of azithromycin and harbored the *mphA* gene as a macrolide resistance determinant. MacConkey agar with azithromycin (32 mg/L) and sodium azide (100 mg/L) was used to differentiate between pink lactose-fermenting *E. coli* colonies and pale non-lactose-fermenting *Shigella* colonies. Recipient *E. coli* J53 can grow only if it receives the AZM resistance factor from donors, as it is susceptible to azithromycin. The identified transconjugants were reconfirmed as *E. coli* using the Vitek-2 system (bioMérieux). The selected and confirmed transconjugants were then subjected to antimicrobial susceptibility tests and plasmid profile analysis.

### Determining plasmid stability

To assess the stability of plasmids in the transconjugant strains, the following experimental procedure was conducted. Initially, the transconjugants were cultured overnight in the presence of antibiotics. The resulting overnight culture was used as a 1% (vol/vol) inoculum in fresh LB broth, resulting in an approximate initial cell density of 5 × 10⁷ CFU/mL. The cultures were subsequently subjected to serial passaging every 24 h for a period of 5 days in antibiotic-free LB broth, maintained at 37°C with shaking at 150 rpm. After each passage, the cultures were serially diluted and plated on LB agar. To evaluate plasmid loss during subculturing, approximately 10 colonies from each culture were randomly selected and screened for the presence of the respective antibiotic resistance genes using PCR.

## Supplementary Material

Reviewer comments

## Data Availability

The sequencing reads used for this study were deposited in the National Center for Biotechnology Information (NCBI) under the BioProject accession number PRJNA1311748.

## SUPPLEMENTAL MATERIAL

File S1 spectrum.02824-25-s0001.docx Step-by-step command workflow for Whole genome sequence (WGS) analysis and Phylogenetic analysis.

Figures S1 to S4 spectrum.02824-25-s0002.pdf Fig. S1: PFGE of representative XDR SS. Fig. S2: Plasmid Profile. Fig. S3: Hybrid assembly. Fig. S4: Phylogenetic workflow.

Table S1 spectrum.02824-25-s0003.xlsx XDR Shigella WGS read QC and accession.

Table S2 spectrum.02824-25-s0004.xlsx List of NCBI accession numbers of all the isolates used as global strains for phylogenetic analysis.

OPEN PEER REVIEW reviewer-comments.pdf An accounting of the reviewer comments and feedback.

ASM does not own the copyrights to Supplemental Material that may be linked to, or accessed through, an article. The authors have granted ASM a non-exclusive, world-wide license to publish the Supplemental Material files. Please contact the corresponding author directly for reuse.

## References

[1] Khalil IA, Troeger C, Blacker BF, Rao PC, Brown A, Atherly DE, Brewer TG, Engmann CM, Houpt ER, Kang G, et al.. 2018. Morbidity and mortality due to *shigella* and enterotoxigenic *Escherichia coli* diarrhoea: the Global Burden of Disease Study 1990-2016. Lancet Infect Dis 18:1229–1240. doi:10.1016/S1473-3099(18)30475-4 30266330 PMC6202441

[2] Taneja N, Mewara A, Kumar A, Verma G, Sharma M. 2012. Cephalosporin-resistant *Shigella flexneri* over 9 years (2001-09) in India. J Antimicrob Chemother 67:1347–1353. doi:10.1093/jac/dks061 22410619

[3] Jain PA, Kulkarni RD, Dutta S, Ganavali AS, Kalabhavi AS, Shetty PC, Shubhada C, Hosamani MA, Appannanavar SB, Hanamaraddi DR. 2020. Prevalence and antimicrobial profile of *Shigella* isolates in a tertiary care hospital of North Karnataka: a 12-year study. Indian J Med Microbiol 38:101–108. doi:10.4103/ijmm.IJMM_20_107 32719216

[4] Ghosh S, Pazhani GP, Chowdhury G, Guin S, Dutta S, Rajendran K, Bhattacharya MK, Takeda Y, Niyogi SK, Nair GB, Ramamurthy T. 2011. Genetic characteristics and changing antimicrobial resistance among *Shigella* spp. isolated from hospitalized diarrhoeal patients in Kolkata, India. J Med Microbiol 60:1460–1466. doi:10.1099/jmm.0.032920-0 21659504

[5] Aggarwal P, Uppal B, Ghosh R, Krishna Prakash S, Chakravarti A, Rajeshwari K. 2016. True prevalence of shigellosis in indian children with acute gastroenteritis: have we been missing the diagnosis? J Res Health Sci 16:11–16. 27061990 PMC7189089

[6] Zaidi MB, Estrada-García T. 2014. *Shigella*: a highly virulent and elusive pathogen. Curr Trop Med Rep 1:81–87. doi:10.1007/s40475-014-0019-6 25110633 PMC4126259

[7] Williams PCM, Berkley JA. 2018. Guidelines for the treatment of dysentery (shigellosis): a systematic review of the evidence. Paediatr Int Child Health 38:S50–S65. doi:10.1080/20469047.2017.1409454 29790845 PMC6021764

[8] Taneja N, Mewara A. 2016. Shigellosis: epidemiology in India. Indian J Med Res 143:565–576. doi:10.4103/0971-5916.187104 27487999 PMC4989829

[9] Ranjbar R, Farahani A. 2019. *Shigella*: antibiotic-resistance mechanisms and new horizons for treatment. Infect Drug Resist 12:3137–3167. doi:10.2147/IDR.S219755 31632102 PMC6789722

[10] Charles H, Prochazka M, Thorley K, Crewdson A, Greig DR, Jenkins C, Painset A, Fifer H, Browning L, Cabrey P, Smith R, Richardson D, Waters L, Sinka K, Godbole G, Outbreak Control Team. 2022. Outbreak of sexually transmitted, extensively drug-resistant *Shigella sonnei* in the UK, 2021-22: a descriptive epidemiological study. Lancet Infect Dis 22:1503–1510. doi:10.1016/S1473-3099(22)00370-X 35809593

[11] Thorley K, Charles H, Greig DR, Prochazka M, Mason LCE, Baker KS, Godbole G, Sinka K, Jenkins C. 2023. Emergence of extensively drug-resistant and multidrug-resistant *Shigella flexneri* serotype 2a associated with sexual transmission among gay, bisexual, and other men who have sex with men, in England: a descriptive epidemiological study. Lancet Infect Dis 23:732–739. doi:10.1016/S1473-3099(22)00807-6 36731481

[12] Lefèvre S, Njamkepo E, Feldman S, Ruckly C, Carle I, Lejay-Collin M, Fabre L, Yassine I, Frézal L, Pardos de la Gandara M, Fontanet A, Weill F-X. 2023. Rapid emergence of extensively drug-resistant *Shigella sonnei* in France. Nat Commun 14:462. doi:10.1038/s41467-023-36222-8 36709320 PMC9883819

[13] Bose P, Chowdhury G, Halder G, Ghosh D, Deb AK, Kitahara K, Miyoshi S-I, Morita M, Ramamurthy T, Dutta S, Mukhopadhyay AK. 2024. Prevalence and changing antimicrobial resistance profiles of *Shigella* spp. isolated from diarrheal patients in Kolkata during 2011-2019. PLoS Negl Trop Dis 18:e0011964. doi:10.1371/journal.pntd.0011964 38377151 PMC10906866

[14] Tian J. 2024. Rural household vulnerability and COVID-19: evidence from India. PLoS One 19:e0301662. doi:10.1371/journal.pone.0301662 38635842 PMC11025904

[15] Dang A, Das M, Gupta I. 2026. COVID ‐19 and the unequal distribution of poverty risks: evidence from urban India. Review Development Economics 30:125–139. doi:10.1111/rode.13268

[16] Gupta A, Das P, Kim D. 2024. The need for localized, socio-economic policy measures for controlling a pandemic: an empirical study of COVID-19 in India. Vikalpa: The Journal for Decision Makers 49:213–229. doi:10.1177/02560909241260234

[17] Malik SS, Mundra S. 2022. Increasing consumption of antibiotics during the COVID-19 pandemic: implications for patient health and emerging anti-microbial resistance. Antibiotics (Basel) 12:45. doi:10.3390/antibiotics12010045 36671246 PMC9855050

[18] Yang X, Li X, Qiu S, Liu C, Chen S, Xia H, Zeng Y, Shi L, Chen J, Zheng J, Yang S, Tian G, Liu G, Yang L. 2024. Global antimicrobial resistance and antibiotic use in COVID-19 patients within health facilities: a systematic review and meta-analysis of aggregated participant data. J Infect 89:106183. doi:10.1016/j.jinf.2024.106183 38754635

[19] Lukose L, Kaur G, M MA, Abraham GA, Khera K, Subeesh VK, Castelino RL, Karanth S, Udyavara Kudru C, Varma M, Miraj SS. 2024. Predictors and patterns of empirical antibiotic therapy and associated outcomes in COVID-19 patients: a retrospective study in a tertiary care facility in South India. Expert Rev Anti Infect Ther 22:333–341. doi:10.1080/14787210.2024.2303019 38189087

[20] Sili U, Tekin A, Bilgin H, Khan SA, Domecq JP, Vadgaonkar G, Segu SS, Rijhwani P, Raju U, Surapaneni KM, Zabolotskikh I, Gomaa D, Goodspeed VM, Ay P. 2024. Early empiric antibiotic use in COVID-19 patients: results from the international VIRUS registry. Int J Infect Dis 140:39–48. doi:10.1016/j.ijid.2023.12.006 38128643 PMC10939992

[21] Cong W, Stuart B, AIhusein N, Liu B, Tang Y, Wang H, Wang Y, Manchundiya A, Lambert H. 2022. Antibiotic use and bacterial infection in COVID-19 patients in the second phase of the SARS-CoV-2 pandemic: a scoping review. Antibiotics (Basel) 11:991. doi:10.3390/antibiotics11080991 35892381 PMC9331316

[22] Sulis G, Batomen B, Kotwani A, Pai M, Gandra S. 2021. Sales of antibiotics and hydroxychloroquine in India during the COVID-19 epidemic: an interrupted time series analysis. PLoS Med 18:e1003682. doi:10.1371/journal.pmed.1003682 34197449 PMC8248656

[23] Ravi G, Chikara G, Bandyopadhyay A, Handu S. 2021. A prospective study to evaluate antimicrobial prescribing pattern among admitted patients in hilly Himalayan region of northern India. J Family Med Prim Care 10:1607–1613. doi:10.4103/jfmpc.jfmpc_1230_20 34123900 PMC8144753

[24] Chakrabarti A, Balaji V, Bansal N, Gopalakrishnan R, Gupta P, Jain A, Kale P, Kapil A, Nath Prasad K, Ray P, Rodrigues C, Walia K. 2025. NAMS task force report on antimicrobial resistance. Ann Natl Acad Med Sci (India) 61:171–209. doi:10.25259/ANAMS_TFR_13_2024

[25] Thanh Duy P, Thi Nguyen TN, Vu Thuy D, Chung The H, Alcock F, Boinett C, Dan Thanh HN, Thanh Tuyen H, Thwaites GE, Rabaa MA, Baker S. 2020. Commensal *Escherichia coli* are a reservoir for the transfer of XDR plasmids into epidemic fluoroquinolone-resistant *Shigella sonnei*. Nat Microbiol 5:256–264. doi:10.1038/s41564-019-0645-9 31959970 PMC6992430

[26] Qiu S, Liu K, Yang C, Xiang Y, Min K, Zhu K, Liu H, Du X, Yang M, Wang L, Sun Y, Zhou H, Mahe M, Zhao J, Li S, Yu D, Hawkey J, Holt KE, Baker S, Yang J, Xu X, Song H. 2022. A *Shigella sonnei* clone with extensive drug resistance associated with waterborne outbreaks in China. Nat Commun 13:7365. doi:10.1038/s41467-022-35136-1 36450777 PMC9709761

[27] Trivett H. 2022. Increase in extensively drug resistant *Shigella sonnei* in Europe. Lancet Microbe 3:e481. doi:10.1016/S2666-5247(22)00160-4 35779565

[28] Choi H, Navarathna DH, Harston BL, Hwang M, Corona B, San Juan MR, Jinadatha C. 2023. Case of extensively drug-resistant *Shigella sonnei* Infection, United States. Emerg Infect Dis 29:1708–1711. doi:10.3201/eid2908.230411 37486233 PMC10370867

[29] Jacqueline C, Carrascoso GR, Gutiérrez-Fernández J, Rangel TV, Goterris L, Valdes FV, Vecilla DF, López ME, Martinez Ruiz MR, Sancho CA, Tanoira RP, Quílez ES, la Rica Martínez A de, Jiménez NG, Salguero CG, Barbera EG, Sánchez Florez MR, Merino FJ, Redondo BS, Guerrero ER, González CS, Herrera-Leon S. 2023. Genetic characterization of extensively drug-resistant *Shigella sonnei* infections, Spain, 2021-2022. Emerg Infect Dis 29:2370–2373. doi:10.3201/eid2911.221746 37877619 PMC10617328

[30] Moreno-Mingorance A, Mir-Cros A, Goterris L, Rodriguez-Garrido V, Sulleiro E, Barberà MJ, Alberny M, Hoyos-Mallecot Y, Descalzo V, Bravo A, Roca-Grande J, Viñado B, Pumarola T, Larrosa MN, González-López JJ. 2023. Increasing trend of antimicrobial resistance in *Shigella* associated with MSM transmission in Barcelona, 2020-21: outbreak of XRD *Shigella sonnei* and dissemination of ESBL-producing *Shigella flexneri*. J Antimicrob Chemother 78:975–982. doi:10.1093/jac/dkad031 36760088 PMC10068420

[31] Mason LCE, Greig DR, Cowley LA, Partridge SR, Martinez E, Blackwell GA, Chong CE, De Silva PM, Bengtsson RJ, Draper JL, Ginn AN, Sandaradura I, Sim EM, Iredell JR, Sintchenko V, Ingle DJ, Howden BP, Lefèvre S, Njamkepo E, Weill F-X, Ceyssens P-J, Jenkins C, Baker KS. 2023. The evolution and international spread of extensively drug resistant *Shigella sonnei*. Nat Commun 14:1983. doi:10.1038/s41467-023-37672-w 37031199 PMC10082799

[32] Surawicz CM. 2007. *Shigella*: a sexually transmitted infection in men who have sex with men. Gastroenterology 133:1737–1738. doi:10.1053/j.gastro.2007.09.044 17983823

[33] Mook P, McCormick J, Bains M, Cowley LA, Chattaway MA, Jenkins C, Mikhail A, Hughes G, Elson R, Day M, Manuel R, Dave J, Field N, Godbole G, Dallman T, Crook P. 2016. ESBL-Producing and macrolide-resistant *Shigella sonnei* infections among men who have sex with men, England, 2015. Emerg Infect Dis 22:1948–1952. doi:10.3201/eid2211.160653 27767929 PMC5088027

[34] Nandi D, Kaur KN, Janardhanan R. 2023. *Shigella* storm and men who have sex with men: a perspective on the Indian context. Indian J Public Health 67:480–481. doi:10.4103/ijph.ijph_1947_21 37929397

[35] Skripai A, Katz A, Lellouche J, Trabelsi O, Sharon N. 2026. Emerging ceftriaxone resistance in paediatric *Shigella sonnei*: a 10-year retrospective study. Acta Paediatr 115:1063–1069. doi:10.1111/apa.70449 41555678

[36] Luzzaro F, Clément M, Principe L, Viaggi V, Bernasconi OJ, Endimiani A. 2019. Characterisation of the first extended-spectrum β-lactamase (ESBL)-producing *Shigella sonnei* clinical isolate in Italy. J Glob Antimicrob Resist 17:58–59. doi:10.1016/j.jgar.2019.03.004 30877056

[37] Campos-Madueno EI, Bernasconi OJ, Moser AI, Keller PM, Luzzaro F, Maffioli C, Bodmer T, Kronenberg A, Endimiani A. 2020. Rapid increase of CTX-M-producing *Shigella sonnei* Isolates in Switzerland due to spread of common plasmids and international clones. Antimicrob Agents Chemother 64:e01057-20. doi:10.1128/AAC.01057-20 32718957 PMC7508577

[38] López-Cerero L, Stolz E, Pulido MR, Pascual A. 2022. Characterization of extended-spectrum β-lactamase-producing *Shigella sonnei* in Spain: expanding the geographic distribution of sequence type 152/CTX-M-27 clone. Antimicrob Agents Chemother 66:e00334-22. doi:10.1128/aac.00334-22 35762798 PMC9295536

[39] Gonzales Rodriguez A, Gonzales Escalante E, Lezameta Abarca L, Saavedra Gutierrez J. 2024. Emergence of lineage III of *Shigella sonnei* ST152 belonging to a high-risk clone harboring the bla_CTX-M-15_ gene in Peru. Rev Argent Microbiol 56:205–209. doi:10.1016/j.ram.2024.02.007 38845247

[40] Leung V, Ritchie G, Stefanovic A, Lee C, Chorlton S, Matic N, Romney MG, Hayden A, Lowe CF. 2025. An outbreak of multidrug-resistant *Shigella flexneri* serotype 2a among people experiencing homelessness in Vancouver. Trop Med Infect Dis 10:120. doi:10.3390/tropicalmed10050120 40423350 PMC12115784

[41] Gaudreau C, Bernaquez I, Pilon PA, Goyette A, Yared N, Bekal S. 2022. Clinical and genomic investigation of an international ceftriaxone- and azithromycin-resistant *Shigella sonnei* cluster among men who have sex with men, Montréal, Canada 2017-2019. Microbiol Spectr 10:e0233721. doi:10.1128/spectrum.02337-21 35647695 PMC9241791

[42] Kharat AA, Makwana N, Kadam DG, Chavan AS, Kulkarni JA, Kharat AS. 2023. Co-existence of extended spectrum β-lactamase and carbapenemase-producing genes from diarrheagenic enteric pathogens isolated in a tertiary care hospital. Acta Biochim Pol 70:69–76. doi:10.18388/abp.2020_6188 36696565

[43] Charles H, Sinka K, Simms I, Baker KS, Godbole G, Jenkins C. 2024. Trends in shigellosis notifications in England, January 2016 to March 2023. Epidemiol Infect 152:e115. doi:10.1017/S0950268824001006 39363593 PMC11450503

[44] Gonzalez-Ferrer S, Shaw B, Fung L, Landovitz RJ, Uslan DZ, Yang S. 2026. Emergence of a novel genotype of extensively drug-resistant *Shigella sonnei* carrying blaDHA-1 in diverse patient populations. Antimicrob Steward Healthc Epidemiol 6:e19. doi:10.1017/ash.2025.10280 41562027 PMC12813715

[45] Muthuirulandi Sethuvel DP, Anandan S, Murugan D, Asokan K, Vasudevan K, Jacob JJ, Walia K, Michael JS, Veeraraghavan B. 2021. Hybrid genome assembly of *Shigella sonnei* reveals the novel finding of chromosomal integration of an IncFII plasmid carrying a *mphA* gene. Access Microbiol 3:000189. doi:10.1099/acmi.0.000189 34151144 PMC8209639

[46] Asad A, Jahan I, Munni MA, Begum R, Mukta MA, Saif K, Faruque SN, Hayat S, Islam Z. 2024. Multidrug-resistant conjugative plasmid carrying mphA confers increased antimicrobial resistance in *Shigella*. Sci Rep 14:6947. doi:10.1038/s41598-024-57423-1 38521802 PMC10960829

[47] Caldera JR, Shaw B, Uslan DZ, Yang S. 2025. Cluster of extensively drug-resistant *Shigella sonnei* carrying bla_CTX-M-15_ in Los Angeles, California, 2023 to 2024. Am J Infect Control 53:524–526. doi:10.1016/j.ajic.2024.12.005 39667596

[48] Seethalakshmi PS, Charity OJ, Giakoumis T, Kiran GS, Sriskandan S, Voulvoulis N, Selvin J. 2022. Delineating the impact of COVID-19 on antimicrobial resistance: an Indian perspective. Sci Total Environ 818:151702. doi:10.1016/j.scitotenv.2021.151702 34798093 PMC8592853

[49] Rahman M, Haque AF, Deeba IM, Ahmed D, Zahidi T, Rimu AH, Akter M, Akter F, Talukder KA. 2017. Emergence of extensively drug-resistant *Shigella sonnei* in Bangladesh. iid 5:1–9. doi:10.13189/iid.2017.050101

[50] Nair GB, Ramamurthy T, Bhattacharya MK, Krishnan T, Ganguly S, Saha DR, Rajendran K, Manna B, Ghosh M, Okamoto K, Takeda Y. 2010. Emerging trends in the etiology of enteric pathogens as evidenced from an active surveillance of hospitalized diarrhoeal patients in Kolkata, India. Gut Pathog 2:4. doi:10.1186/1757-4749-2-4 20525383 PMC2901208

[51] CLSI. 2020. Performance Standards for Antimicrobial Susceptibility Testing. *In* CLSI supplement M100, 30th ed. Clinical and Laboratory Standards Institute, Wayne, PA.

[52] Phuc Nguyen MC, Woerther P-L, Bouvet M, Andremont A, Leclercq R, Canu A. 2009. *Escherichia coli* as reservoir for macrolide resistance genes. Emerg Infect Dis 15:1648–1650. doi:10.3201/eid1510.090696 19861064 PMC2866414

[53] Halder G, Chaudhury BN, Mandal S, Denny P, Sarkar D, Chakraborty M, Khan UR, Sarkar S, Biswas B, Chakraborty A, Maiti S, Dutta S. 2024. Whole genome sequence-based molecular characterization of blood isolates of carbapenem-resistant *Enterobacter cloacae* complex from ICU patients in Kolkata, India, during 2017-2022: emergence of phylogenetically heterogeneous *Enterobacter hormaechei* subsp. *xiangfangensis* Microbiol Spectr 12:e0352923. doi:10.1128/spectrum.03529-23 38385742 PMC10986559

[54] Croucher NJ, Page AJ, Connor TR, Delaney AJ, Keane JA, Bentley SD, Parkhill J, Harris SR. 2015. Rapid phylogenetic analysis of large samples of recombinant bacterial whole genome sequences using Gubbins. Nucleic Acids Res 43:e15. doi:10.1093/nar/gku1196 25414349 PMC4330336

[55] Minh BQ, Schmidt HA, Chernomor O, Schrempf D, Woodhams MD, von Haeseler A, Lanfear R. 2020. IQ-TREE 2: new models and efficient methods for phylogenetic inference in the genomic era. Mol Biol Evol 37:1530–1534. doi:10.1093/molbev/msaa015 32011700 PMC7182206

[56] Kalyaanamoorthy S, Minh BQ, Wong TKF, von Haeseler A, Jermiin LS. 2017. ModelFinder: fast model selection for accurate phylogenetic estimates. Nat Methods 14:587–589. doi:10.1038/nmeth.4285 28481363 PMC5453245

