# Supplementary material for: Escalation of CTX-M-producing extensively drug-resistant Shigella spp. in Kolkata, India, following the COVID-19 pandemic
Source: Microbiol Spectr. 2026 Jul 24;14(9):e02824-25. doi: 10.1128/spectrum.02824-25 (PMC13532320; doi:10.1128/spectrum.02824-25)
Supplement: Supplementary file 1 [file reviewer-comments.pdf]

AMERICAN SOCIETY FOR MICROBIOLOGY | Microbiology Spectrum

Escalation of CTX-M-Producing Extensively Drug-Resistant Shigella spp. in Kolkata, India, Following the COVID-19 Pandemic

Puja Bose, Gourab Halder, Pratanu Kayet, Goutam Chowdhury, Surajit Basak, Deboleena Roy, Daisuke Imamura, SHIN-ICHI MIYOSHI, Masatomo Morita, Thandavarayan Ramamurthy, Hemanta Koley, Santasabuj Das, and Asish Mukhopadhyay

Corresponding Author(s): Asish Mukhopadhyay, ICMR - National Institute for Research in Bacterial Infections

Review Timeline:

Submission Date:	September 8, 2025
Editorial Decision:	January 19, 2026
Revision Received:	March 19, 2026
Editorial Decision:	April 8, 2026
Revision Received:	April 16, 2026
Accepted:	April 16, 2026

Editor: Reema Singh

Reviewer(s): Disclosure of reviewer identity is with reference to reviewer comments included in decision letter(s). The following individuals involved in review of your submission have agreed to reveal their identity: Sadjia Bekal (Reviewer #3)

Transaction Report:

(Note: With the exception of the correction of typographical or spelling errors that could be a source of ambiguity, letters and reports are not edited. The original formatting of letters and referee reports may not be reflected in this compilation.)

DOI: https://doi.org/10.1128/spectrum.02824-25

Re: Spectrum02824-25 (Escalation of CTX-M-Producing Extensively Drug-Resistant Shigella spp. in Kolkata, India, Following the COVID-19 Pandemic)

Dear Dr. Asish K. Mukhopadhyay:

Thank you for the privilege of reviewing your work. Below you will find my comments, instructions from the Spectrum editorial office, and the reviewer comments.

The study by Bose et al. investigates the alarming rise of extensively drug-resistant (XDR) Shigella species in Kolkata, India, identifying a significant shift in antimicrobial resistance (AMR) patterns following the COVID-19 pandemic. By analyzing clinical isolates from pediatric diarrheal cases between 2021 and 2023 and comparing them to a 2013-2019 baseline, the authors document a sharp escalation in resistance to critical oral antibiotics, including ciprofloxacin, ceftriaxone, and azithromycin. Overall, the study is well presented and written.

The reviewers and I see significant value in your genomic analysis of XDR Shigella. At the same time, the manuscript requires a more rigorous description of the study limitations and a substantial shift in data interpretation before it can be considered for publication

1. While the surveillance data is valuable, the manuscript currently reads as a report of findings. In your revision, please strengthen the Discussion by contextualizing these findings within the socio-economic and healthcare landscape of post-pandemic India. Specifically, discuss how the shifts in antibiotic prescribing patterns during 2020-2021 may have created a selective sweep for these specific XDR lineages

2. Please ensure the study design is accurately labeled. Since this involves the collection of clinical isolates at the point of care, 'prospective surveillance' or 'cross-sectional analysis' is more appropriate than 'prospective study.' Mislabeling the study design undermines the high-quality genomic work you have presented.

3. The reviewer highlights the importance of your transconjugation experiments. Please expand on the clinical implications of the plasmid-mediated spread. Specifically, address the stability of the blaCTX-M-15 and mphA genes, if these are moving from plasmids to the chromosome (as suggested by your Nanopore data), what does this mean for the long-term persistence of XDR Shigella even if antibiotic pressure is reduced?

4. A formal 'Limitations' section must be added. You must explicitly discuss the potential for selection bias in your sampling and how the lack of a non-diarrheal control group affects the generalizability of your results regarding gut microbiota carriage.

5. While the BioProject ID (PRJNA1311748) is noted, please ensure that all individual SRA accession numbers for the 38 sequenced isolates are clearly provided (e.g., in a supplemental table) and that the data is set to 'Public' status before resubmission. Additionally, please review the manuscript for typographical errors; specifically, ensure all gene names (e.g., blaCTX-M-15, mphA) use standard Latin characters and are consistently italicized.

6. To ensure reproducibility and address reviewer concerns regarding phylogenetic assumptions, please provide a more detailed, step-by-step description of the bioinformatics pipeline. Specifically, include the versions and key parameters/settings used for SNP calling (Snippy), recombination filtering (Gubbins), and the specific criteria used to select the 1,888 global reference genomes. This will provide necessary transparency for the large-scale comparative analysis.

Please return the manuscript within 60 days; if you cannot complete the modification within this time period, please contact me. If you do not wish to modify the manuscript and prefer to submit it to another journal, notify me immediately so that the manuscript may be formally withdrawn from consideration by Spectrum.

Revision Guidelines
To submit your modified manuscript, log into the submission site at https://spectrum.msubmit.net/cgi-bin/main.plex. Go to Author Tasks and click the appropriate manuscript title to begin. The information you entered when you first submitted the paper will be displayed; update this as necessary. Note the following requirements:

• Upload point-by-point responses to the issues raised by the reviewers in a file named "Response to Reviewers," NOT in your cover letter.
• Upload a compare copy of the manuscript (without figures) as a "Marked-Up Manuscript" file.
• Upload a clean .DOC/.DOCX version of the revised manuscript and remove the previous version.
• Each figure must be uploaded as a separate, editable, high-resolution file (TIFF or EPS preferred), and any multipanel figures must be assembled into one file.
• Any supplemental material intended for posting by ASM should be uploaded with their legends separate from the main manuscript. You can combine all supplemental material into one file (preferred) or split it into a maximum of 10 files with all

associated legends included.

For complete guidelines on revision requirements, see our Submission and Review Process webpage. Submission of a paper that does not conform to guidelines may delay acceptance of your manuscript.

Data availability: ASM policy requires that data be available to the public upon online posting of the article, so please verify all links to sequence records, if present, and make sure that each number retrieves the full record of the data. If a new accession number is not linked or a link is broken, provide Spectrum production staff with the correct URL for the record. If the accession numbers for new data are not publicly accessible before the expected online posting of the article, publication may be delayed; please contact production staff (Spectrum@asmusa.org) immediately with the expected release date.

Publication Fees: For information on publication fees and which article types are subject to charges, visit our website. If your manuscript is accepted for publication and any fees apply, you will be contacted separately about payment during the production process; please follow the instructions in that e-mail. Arrangements for payment must be made before your article is published.

ASM Membership: Corresponding authors may join or renew ASM membership to obtain discounts on publication fees. Need to upgrade your membership level? Please contact Customer Service at Service@asmusa.org.

The ASM Journals program strives for constant improvement in our submission and publication process. Please tell us how we can improve your experience by taking this quick Author Survey.

Thank you for submitting your paper to Spectrum.

Sincerely,
Reema Singh
Editor
Microbiology Spectrum

Reviewer #2 (Comments for the Author):

Thank you for this interesting report. I would ask that you please list the limitations of the study in the discussion beginning with the sentence "there are several limitations to this study......" including selection bias as the cases presented were all from isolates that were obtained from patients with diarrhea. Could the presence of resistance genes among healthy asymptomatic volunteers (but carriers) be determined probing for ARG's among those positive for shigella? The title could be changed from "Escalation of CTX-M-Producing Extensively Drug-Resistant Shigella spp. in 1 Kolkata, India, Following the COVID-19 Pandemic" to "Post COVID-19 pandemic surveillance of drug resistant Shigella spp. in Kolkata ,India". I do not think the term "extensively" is warranted here. In the introduction, it would be advisable to state the percentage of cases of childhood diarrhea that are actually due to Shigella, which by my reading is less than 25%, obviously still significant. Since India has among the highest rates of AMR worldwide and because antibiotics are obtainable over-the-counter (OTC), this might be mentioned in the discussion.

Minor

Line 192 similarto should be similar to
Line 193 "showed MIC" should be showed an MIC of......
Line 209... Prevalence of .... Should be The prevalence of ....

Reviewer #3 (Comments for the Author):

The study raises a public health issue that began to emerge in the 2010s with the development of azithromycin resistance, followed by the acquisition of other resistances, leading to the XDR phenotype in Shigella, but most often linked to the MSM community in published studies. The study is well-designed, but lacks certain elements to make it optimal, and some aspects are not relevant to this publication.
Major comments
1. The title, as worded, seems to imply a link between the emergence of XDR strains and the pre- and post-COVID periods. If this is the case, the authors should elaborate on their hypotheses; otherwise, they should remove it from the title so that a quick reading of the article in NCBI does not infer this link.
2. Line 60: Did you compare India strains to international ones, and were similar to England ones only please add this information.
3. As a reader, I was expecting a full interpretation of PFGE patterns in the context of this study. The aim and results interpretation? knowing also the limit of PFGE for phylogenetic links confirmation\infirmation

4. Authors should explain the criteria to choose representative strains for whole genome sequencing, from PFGE patterns
5. Figure S2 is not relevant for this study.
6. Authors should add parameters quality of the genomes uses in this study for short reads (coverage, N50, number of contigs..etc..) as these parameters can affect the quality and therefore interpretation.
7. Phylogenetic analysis using WGS should be discussed using for phylogenetic criteria for inclusion/exclusion from a genomic cluster.
8. The fact that these XDR strains are mostly found in pediatric population, while they are associated to MSM worldwide, is of a high concern for public health and treatment for infected children. I encourage authors to more highlight this finding.

Minor comments

1. Line 42: please add the number of the ceftriaxone-resistant S. sonnei to better appreciate the proportion of those XDR ones
2. Line 71: Please add the value of this very low infectious dose and reference
3. References 5 and 7 are missing information. Please add date, year...etc

The study raises a public health issue that began to emerge in the 2010s with the development of azithromycin resistance, followed by the acquisition of other resistances, leading to the XDR phenotype in Shigella, but most often linked to the MSM community in published studies. The study is well-designed, but lacks certain elements to make it optimal, and some aspects are not relevant to this publication.

Major comments

1. The title, as worded, seems to imply a link between the emergence of XDR strains and the pre- and post-COVID periods. If this is the case, the authors should elaborate on their hypotheses; otherwise, they should remove it from the title so that a quick reading of the article in NCBI does not infer this link.
2. Line 60: Did you compare India strains to international ones, and were similar to England ones only please add this information.
3. As a reader, I was expecting a full interpretation of PFGE patterns in the context of this study. The aim and results interpretation? knowing also the limit of PFGE for phylogenetic links confirmation\infirmation
4. Authors should explain the criteria to choose representative strains for whole genome sequencing, from PFGE patterns
5. Figure S2 is not relevant for this study.
6. Authors should add parameters quality of the genomes uses in this study for short reads (coverage, N50, number of contigs..etc..) as these parameters can affect the quality and therefore interpretation.
7. Phylogenetic analysis using WGS should be discussed using for phylogenetic criteria for inclusion/exclusion from a genomic cluster.
8. The fact that these XDR strains are mostly found in pediatric population, while they are associated to MSM worldwide, is of a high concern for public health and treatment for infected children. I encourage authors to more highlight this finding.

Minor comments

1. Line 42: please add the number of the ceftriaxone-resistant S. sonnei to better appreciate the proportion of those XDR ones
2. Line 71: Please add the value of this very low infectious dose and reference
3. References 5 and 7 are missing information. Please add date, year…etc

Title of the Manuscript: “Escalation of CTX-M Producing Extensively Drug-Resistant Shigella spp. in Kolkata, India, Following COVID-19 Pandemic (Ref: Spectrum2824-25)

Response to the comments from the authors
We are grateful to the editor and reviewers for their insightful comments, which we have critically considered to revise the manuscript. Following are the detailed point-by-point response.

# In house Editorial comment:

Comment 1: While the surveillance data is valuable, the manuscript currently reads as a report of findings. In your revision, please strengthen the Discussion by contextualizing these findings within the socio-economic and healthcare landscape of post-pandemic India. Specifically, discuss how the shifts in antibiotic prescribing patterns during 2020-2021 may have created a selective sweep for these specific XDR lineages.

Response: We appreciate the editor’s insightful comment to better contextualize our findings within the broader socio-economic and healthcare framework of post-pandemic India. In response, we have substantially revised the Discussion section focusing on findings within the socio-economic and healthcare landscape of post-pandemic India. We have also specifically discussed how the shifts in antibiotic prescribing patterns occurred during 2020-2021. Accordingly, we have included the appropriate statements in the Discussion section of the revised manuscript (Lines 208-234) with new references. We believe this expanded discussion enhances the interpretation of our findings and aligns with evolving evidence on how the pandemic reshaped infectious disease dynamics in low- and middle-income settings.

Comment 2: Please ensure the study design is accurately labeled. Since this involves the collection of clinical isolates at the point of care, 'prospective surveillance' or 'cross-sectional analysis' is more appropriate than 'prospective study.' Mislabeling the study design undermines the high-quality genomic work you have presented.

Response: We thank the reviewer for this important clarification regarding study design terminology. We agree that the term “prospective study” may not precisely reflect the nature of isolate collection in our work. Accordingly, we have revised the manuscript throughout to describe the work as “prospective laboratory surveillance.” These changes have been made in the abstract to ensure an accurate epidemiological representation of the study design (Lines 35-36).

Comment 3: The reviewer highlights the importance of your transconjugation experiments. Please expand on the clinical implications of the plasmid-mediated spread. Specifically, address the stability of the *bla*$_{CTX-M-15}$ and *mphA* genes, if these are moving from plasmids to the chromosome (as suggested by your Nanopore data), what does this mean for the long-term persistence of XDR *Shigella* even if antibiotic pressure is reduced?

Response: We thank the reviewer for highlighting the important clinical implications of plasmid-mediated resistance and the potential chromosomal integration suggested by our Nanopore sequencing data. We have expanded the Results (188-195), Discussion (Lines 293-312) and Methods (496-505) to address these aspects in greater depth. We believe this clarifies how plasmid stability and chromosomal integration contribute to the long-term persistence and epidemiological success of XDR *Shigella* in the post-pandemic setting.

Comment 4: A formal 'Limitations' section must be added. You must explicitly discuss the potential for selection bias in your sampling and how the lack of a non-diarrheal control group affects the generalizability of your results regarding gut microbiota carriage.

Response: We thank the reviewer for this important suggestion. We have now added a dedicated "Limitations" section to the manuscript (Lines 327-369). In this section, we explicitly discuss the potential for selection bias arising from hospital-based sampling of diarrheal cases and clarify that the absence of a non-diarrheal control group limits the generalization of our findings to community-level gut microbiota carriage of resistance determinants along with other drawbacks of our study. These additions help to appropriately contextualize the scope and interpretation of our results while maintaining the focus on clinically relevant *Shigella* infections.

Comment 5: While the BioProject ID (PRJNA1311748) is noted, please ensure that all individual SRA accession numbers for the 38 sequenced isolates are clearly provided (e.g., in a supplemental table) and that the data is set to 'Public' status before resubmission. Additionally, please review the manuscript for typographical errors; specifically, ensure all gene names (e.g., *bla*$_{CTX-M-15}$, *mphA*) use standard Latin characters and are consistently italicized.

Response: We thank the reviewer for this important comment regarding data transparency and nomenclature consistency. All individual SRA accession numbers for the 23 sequenced isolates have now been compiled in a newly added Supplementary Table (Table S1), with

corresponding isolate identifiers clearly indicated. In addition, the BioProject PRJNA1311748 and all associated SRA records have been verified and are set to "Public" status prior to resubmission.

We have also carefully reviewed the entire manuscript for typographical inconsistencies. All antimicrobial resistance gene names (e.g., $bla_{CTX-M-15}$, *mphA*) have been uniformly followed with Latin characters and consistent formatting in accordance with accepted gene nomenclature conventions. Corrections have been made throughout the main text, tables, and supplementary materials.

Comment 6: To ensure reproducibility and address reviewer concerns regarding phylogenetic assumptions, please provide a more detailed, step-by-step description of the bioinformatics pipeline. Specifically, include the versions and key parameters/settings used for SNP calling (Snippy), recombination filtering (Gubbins), and the specific criteria used to select the 1,888 global reference genomes. This will provide necessary transparency for the large-scale comparative analysis.

Response: Thank you for this valuable suggestion. To improve transparency and ensure reproducibility of the phylogenetic analysis, we have revised the Bioinformatics and Phylogenetic Analysis section of the manuscript to include a detailed, step-by-step description of the pipeline. Specifically, we have added the software versions and key parameters/settings used for SNP calling (Snippy) and recombination filtering (Gubbins). In addition, we have clarified the criteria used for selecting the 1,888 global reference genomes, including the sources and inclusion parameters applied during dataset curation. Furthermore, to provide a clearer overview of the analytical workflow, we have included a complete phylogenetic analysis pipeline diagram, which is now presented as a supplementary figure (**Fig. S3**). These additions have been incorporated in the revised Methods section (Lines 440-484) to enhance methodological transparency and reproducibility.

Reviewer #2:

Major Revision

Comment: Thank you for this interesting report. I would ask that you please list the limitations of the study in the discussion beginning with the sentence "there are several limitations to this study......" including selection bias as the cases presented were all from isolates that were obtained from patients with diarrhea.

Could the presence of resistance genes among healthy asymptomatic volunteers (but carriers) be determined probing for ARG's among those positive for *Shigella*? The title could be changed from "Escalation of CTX-M-Producing Extensively Drug-Resistant *Shigella* spp. in 1 Kolkata, India, Following the COVID-19 Pandemic" to "Post COVID-19 pandemic surveillance of drug resistant *Shigella* spp. in Kolkata, India". I do not think the term "extensively" is warranted here. In the introduction, it would be advisable to state the percentage of cases of childhood diarrhea that are actually due to Shigella, which by my reading is less than 25%, obviously still significant. Since India has among the highest rates of AMR worldwide and because antibiotics are obtainable over-the-counter (OTC), this might be mentioned in the discussion.

Response:

- Thank you for the suggestions. As mentioned earlier, we have now added a dedicated “Limitations” section to the manuscript (Lines 327-369).
- We thank the reviewer for this insightful suggestion. The assessment of antimicrobial resistance genes (ARGs) among asymptomatic *Shigella* carriers in the community is indeed highly relevant for understanding the potential silent reservoir and community-level dissemination of resistance determinants. However, the present study was designed as a hospital-based surveillance study focused on clinically presenting cases, and did not include parallel community-based sampling or case-control screening of healthy volunteers. Consequently, evaluation of ARG carriage among asymptomatic individuals was beyond the scope of this investigation. We agree that such an approach would provide valuable insight into the extent of community circulation of resistance determinants and represent an important direction for future epidemiological studies. Thus, we have added a para in the limitation section pointing to this issue (Lines 328-334).
- We appreciate the reviewer’s suggestion regarding the modification of the title. However, we reverently wish to retain the term “Extensively Drug-Resistant (XDR)” as it accurately reflects the resistance profile observed in our isolates and carries important clinical and epidemiological implications. The isolates characterized in this study demonstrated resistance to all first-line and alternative drugs recommended for shigellosis management, including third-generation cephalosporins, ciprofloxacin, azithromycin, co-trimoxazole,

and ampicillin. This resistance pattern fulfils the widely accepted definition of XDR, wherein organisms remain susceptible to very limited or no standard therapeutic options (https://www.cdc.gov/han/2023/han00486.html). The use of the term "XDR" is particularly relevant in the context of *Shigella*, as recent global reports have highlighted the emergence of XDR strains, and currently there are no clear CDC or WHO therapeutic guidelines for managing infections caused by such isolates (doi: 10.3201/eid2908.230411; doi: 10.3201/eid2911.221746; doi: 10.1038/s41467-023-36222-8; doi: 10.1093/ofid/ofaf695.778). To our knowledge, this represents one of the earliest reports documenting the emergence of CTX-M-producing XDR *Shigella* spp. from India in the post-COVID period.

Furthermore, a significant finding of our study is the temporal escalation of these XDR isolates following the COVID-19 pandemic, likely associated with altered antibiotic prescribing practices. Removing the term "extensively" from the title would under represent the severity and novelty of the findings. We therefore propose retaining the original title, as it more accurately conveys the clinical significance and emerging public health concerns highlighted by this study.

- In the introduction section, we have added that "but a recent study from India has revealed that the actual prevalence of shigellosis was 18.3% in childhood diarrhea." (Lines 71-72).
- We have revised the discussion part including the suggestions (Lines 197-326)

Minor Revision

Comment 1: Line 192 similarto should be similar to

Response: This typo has been corrected.

Comment 2: Line 193 "showed MIC" should be showed an MIC of......

Response: This suggested correction has been made.

Comment 3: Line 209... Prevalence of .... Should be The prevalence of ....

Response: This suggested correction has been made.

Reviewer #3:

Major Revision

Comment 1: The title, as worded, seems to imply a link between the emergence of XDR strains and the pre- and post-COVID periods. If this is the case, the authors should elaborate on their hypotheses; otherwise, they should remove it from the title so that a quick reading of the article in NCBI does not infer this link.

Response: We thank the reviewer for the valuable suggestion. A widespread misconception that prevails among the common masses is that antibiotics like azithromycin, amoxicillin, amoxiclav, etc., are effective in treating COVID-19. Additionally, suspected COVID-19 cases are reported to use antibiotics. These lead to rapid resistance gain against the macrolide and third-generation cephalosporin classes. Our study hypothesized that irrational use of these antibiotics will positively change the AMR profile of circulating strains. We have revised the discussion in part by including this hypothesis (Lines 208-234).

Comment 2: Line 60: Did you compare India strains to international ones, and were similar to England ones only please add this information.

Response: Thank you for this helpful comment. Yes, we compared the Kolkata isolates with a global collection of international genomes included in our phylogenetic analysis. The results indicated that the Kolkata isolates clustered closely with strains previously reported from England, suggesting genetic similarity within this lineage. We have now clarified this point in the manuscript. The corresponding sentence has been revised in Line 59-61 to explicitly state that "When compared with a global dataset of international genomes, the Kolkata XDR isolates were found to cluster closely with strains reported from England".

Comment 3: As a reader, I was expecting a full interpretation of PFGE patterns in the context of this study. The aim and results interpretation? knowing also the limit of PFGE for phylogenetic links confirmation\information.

Response: The authors are grateful for this suggestion, as WGS is a cost-effective process the authors have undergone through PFGE to eliminate selection of the clonal isolates for the downstream process. PFGE analysis showed that strains from distant lineage are chosen as representative strains for the WGS. A line explaining these selection criteria is added in the result section (Line 400).

Comment 4: Authors should explain the criteria to choose representative strains for whole genome sequencing, from PFGE patterns.

Response: We thank the reviewer for this important suggestion. The criteria for selecting representative isolates for whole genome sequencing have been explained in the revised manuscript.

Representative strains were selected based on their PFGE clustering patterns to confirm the genetic diversity present in the collection. Specifically, isolates were chosen from different PFGE clusters and sub-clusters to ensure that the sequenced strains reflected the major circulating lineages. In addition to PFGE diversity, we carefully considered epidemiological and phenotypic characteristics, including patient demographic information (age, sex, and year of isolation) and the AMR profiles, in order to include isolates representing different resistance patterns and temporal distribution. This strategy allowed us to obtain a representative subset of *Shigella* isolates that reflects both the genotypic diversity and epidemiological characteristics of the overall isolate collection. The description of this selection strategy has been included in the Methods section of the revised manuscript (Lines 410-414).

Comment 5: Figure S2 is not relevant for this study.

Response: We thank the reviewer for this comment. We agree that inclusion of both antibiogram data and plasmid profiling in the original Figure S2 may have lessened its clarity. The antibiogram component has therefore been removed, as this data is described in the main text. However, we have retained the plasmid profiling panel because it directly supports the transconjugation results. The plasmid banding patterns in donor and transconjugant strains provide experimental evidence for plasmid-mediated transfer of resistance determinants and complement the genomic findings. To improve clarity, we have revised the figure legend. We believe the revised figure now clearly supports the mechanistic interpretation of plasmid-mediated resistance transfer.

Comment 6: Authors should add parameters quality of the genomes uses in this study for short reads (coverage, N50, number of contigs etc..) as these parameters can affect the quality and therefore interpretation.

Response: We thank the reviewer for this important suggestion. To ensure transparency and robustness of our genomic analyses, we have now included a dedicated subsection in the

Methods describing the criteria used to assess genome assembly quality. In addition, we have provided detailed assembly metrics for all isolates, including coverage, N50, L50, number of contigs, genome size, and GC content in Supplementary Table S1. All genomes included in the study met the predefined quality thresholds, ensuring reliability of the downstream phylogenetic and comparative genomic interpretations.

Comment 7: Phylogenetic analysis using WGS should be discussed using for phylogenetic criteria for inclusion/exclusion from a genomic cluster.

Response: The authors are thankful for pointing out this important note. We have added "We searched the NCBI database (www.ncbi.nlm.nih.gov) for *S. flexneri* genomes satisfying the following criteria: (i) presence of the *ctxM, sul* and *dfr* gene variant, (ii) mutation in the quinolone resistance-determining region (QRDR) or *qnr* genes. As a result, 297 *S. flexneri* genomes were retrieved. Similarly, we also searched the NCBI database for *S. sonnei* genomes containing *mphA* and/or *ermB*, as well as the presence of the *ctxM* gene variant. In this search, we retrieved 1,888 *S. sonnei* genomes. Therefore, 1,888 *S. sonnei* (Table S2.xlsx) and 297 *S. flexneri* (Table S2.xlsx) downloaded genomes were used in the phylogenetic analysis" (Lines 440-447).

Comment 8: The fact that these XDR strains are mostly found in pediatric population, while they are associated to MSM worldwide, is of a high concern for public health and treatment for infected children. I encourage authors to more highlight this finding.

Response: We thank the reviewer for highlighting this important epidemiological implication of our findings. We agree that the predominance of XDR *Shigella* isolates in the pediatric population, contrasting with the globally reported MSM-associated transmission of XDR *S. sonnei*, represents a significant and concerning shift in transmission dynamics. We have now expanded the discussion to explicitly emphasize this observation (Lines 250-264). In support of this, we have incorporated evidence from a recent 10-year retrospective study demonstrating a sharp rise in ceftriaxone resistance in *S. sonnei* (from 3.2% in 2019 to 78% in 2021), which reflects the rapid expansion of XDR lineages in the region (DOI: 10.1111/apa.70449). We further discuss how increased acquisition of resistance determinants among pediatric circulating strains poses a serious therapeutic and public health challenge. These additions strengthen the contextual interpretation of our genomic findings.

Minor Revision

Comment 1: Line 42: please add the number of the ceftriaxone-resistant S. sonnei to better appreciate the proportion of those XDR ones.

Response: We thank the reviewer for this helpful suggestion. The number of ceftriaxone-resistant *S. sonnei* isolates has now been added in Line 42 to better contextualize the proportion of XDR isolates.

Comment 2: Line 71: Please add the value of this very low infectious dose and reference.

Response: We thank the reviewer for this valuable suggestion. The specific value of the low infectious dose, along with an appropriate reference, has now been added in Line 73.

Comment 3: References 5 and 7 are missing information. Please add date, year...etc

Response: We thank the reviewer for pointing this out. The references were revised and missing bibliographic details (year, date, and other required information) have now been added (Lines 553-554, 556-557).

Re: Spectrum02824-25R1 (Escalation of CTX-M-Producing Extensively Drug-Resistant Shigella spp. in Kolkata, India, Following the COVID-19 Pandemic)

Dear Dr. Asish Kumar Mukhopadhyay:

Thank you for the privilege of reviewing your work. Below you will find my comments, instructions from the Spectrum editorial office, and the reviewer comments.

Thank you for the revised manuscript. The manuscript is ready for publication. I have a few minor comments that I believe would improve clarity and readability for readers:

1. The manuscript describes the work as "prospective laboratory surveillance." The 2021-2023 isolate collection was indeed prospective. The 2013-2019 data are included in Figure 1 and Table 1 for historical comparison only, based on a previous published study. I recommend clarifying in the abstract and methods that the current study is based on prospective laboratory surveillance (2021-2023), and that earlier pre-pandemic data are cited for context. This will accurately represent the study design and avoid potential confusion for readers.

2. Please clarify whether the 2013-2019 (pre-pandemic) isolates are included only in Figure 1 and Table 1 for historical comparison, or if they are also represented in other figures or analyses. This will help readers distinguish between data from the current prospective study and historical data.

3. The authors used assembled genomes of 1,888 S. sonnei and 297 S. flexneri retrieved from NCBI for SNP-based phylogenetic analysis. Since these assemblies were generated using different sequencing technologies and pipelines, differences in assembly quality could impact SNP identification and tree topology. I recommend including a statement in the limitations section acknowledging this potential source of variability.

4. The manuscript now includes a detailed description of the bioinformatics pipeline and key parameters for SNP calling, recombination filtering, and genome selection. To further enhance reproducibility, I recommend providing the actual step-by-step commands or scripts, either as a supplementary file or in a public repository (e.g., GitHub), for the WGS and phylogenetic analysis steps. This would allow other researchers to replicate the analysis and is considered best practice for large-scale genomic studies.

5. Please provide high-quality figures. For example, the text in Figure 3 is difficult to read and would benefit from improved resolution or larger fonts.

Please return the manuscript within 60 days; if you cannot complete the modification within this time period, please contact me. If you do not wish to modify the manuscript and prefer to submit it to another journal, notify me immediately so that the manuscript may be formally withdrawn from consideration by Spectrum.

Revision Guidelines
To submit your modified manuscript, log into the submission site at https://spectrum.msubmit.net/cgi-bin/main.plex. Go to Author Tasks and click the appropriate manuscript title to begin. The information you entered when you first submitted the paper will be displayed; update this as necessary. Note the following requirements:

• Upload point-by-point responses to the issues raised by the reviewers in a file named "Response to Reviewers," NOT in your cover letter.
• Upload a compare copy of the manuscript (without figures) as a "Marked-Up Manuscript" file.
• Upload a clean .DOC/.DOCX version of the revised manuscript and remove the previous version.
• Each figure must be uploaded as a separate, editable, high-resolution file (TIFF or EPS preferred), and any multipanel figures must be assembled into one file.
• Any supplemental material intended for posting by ASM should be uploaded with their legends separate from the main manuscript. You can combine all supplemental material into one file (preferred) or split it into a maximum of 10 files with all associated legends included.

For complete guidelines on revision requirements, see our Submission and Review Process webpage. Submission of a paper that does not conform to guidelines may delay acceptance of your manuscript.

Data availability: ASM policy requires that data be available to the public upon online posting of the article, so please verify all links to sequence records, if present, and make sure that each number retrieves the full record of the data. If a new accession number is not linked or a link is broken, provide Spectrum production staff with the correct URL for the record. If the accession numbers for new data are not publicly accessible before the expected online posting of the article, publication may be delayed; please contact production staff (Spectrum@asmusa.org) immediately with the expected release date.

Publication Fees: For information on publication fees and which article types are subject to charges, visit our website. If your manuscript is accepted for publication and any fees apply, you will be contacted separately about payment during the production process; please follow the instructions in that e-mail. Arrangements for payment must be made before your article is published.

ASM Membership: Corresponding authors may join or renew ASM membership to obtain discounts on publication fees. Need to upgrade your membership level? Please contact Customer Service at Service@asmusa.org.

The ASM Journals program strives for constant improvement in our submission and publication process. Please tell us how we can improve your experience by taking this quick Author Survey.

Thank you for submitting your paper to Spectrum.

Sincerely,
Reema Singh
Editor
Microbiology Spectrum

Reviewer #2 (Comments for the Author):

thank you for the revisions

Reviewer #3 (Comments for the Author):

Thanks for addressing all comments

Title of the Manuscript: "Escalation of CTX-M Producing Extensively Drug-Resistant *Shigella* spp. in Kolkata, India, Following COVID-19 Pandemic (Ref: Spectrum2824-25)

Response to the in house Editorial comment: We are grateful to the editor for the insightful comments, which we have critically considered to revise the manuscript. Following are the detailed point-by-point response.

#1.**Comment:** The manuscript describes the work as "prospective laboratory surveillance." The 2021-2023 isolate collection was indeed prospective. The 2013-2019 data are included in Figure 1 and Table 1 for historical comparison only, based on a previous published study. I recommend clarifying in the abstract and methods that the current study is based on prospective laboratory surveillance (2021-2023), and that earlier pre-pandemic data are cited for context. This will accurately represent the study design and avoid potential confusion for readers.

Response: We thank the reviewer for this important suggestion. We agree that the description of the study as "prospective laboratory surveillance" should specifically refer to the 2021–2023 isolate collection, which represents the primary dataset analyzed in the present work.

In response, we have revised the Abstract (Line: 35-38) and Materials and Methods (Line: 383-386) sections to clarify that the current study is based on prospective laboratory surveillance conducted during 2021–2023. We further specify that the 2013–2019 data presented in **Figure 1** and **Table 1** were included exclusively for pre-pandemic historical comparison and were obtained from our previously published study. We believe this revision improves the accuracy of the study design description and minimizes potential ambiguity for readers.

#2.**Comment:** Please clarify whether the 2013-2019 (pre-pandemic) isolates are included only in Figure 1 and Table 1 for historical comparison, or if they are also represented in other figures or analyses. This will help readers distinguish between data from the current prospective study and historical data.

Response: We thank the reviewer for this helpful suggestion. We have clarified that the 2013–2019 pre-pandemic isolates were included solely for historical comparison in **Figure 1** and **Table 1** and were obtained from our previously published study.

These historical isolates were not included in any other figures, analyses, or conclusions of the current prospective study, which is based exclusively on isolates collected during 2021–2023.

This clarification has now been incorporated into the Materials and Methods section (Line: 383-386) to clearly distinguish the historical comparator dataset from the primary prospective surveillance dataset.

#3.**Comment:** The authors used assembled genomes of 1,888 S. sonnei and 297 S. flexneri retrieved from NCBI for SNP-based phylogenetic analysis. Since these assemblies were generated using different sequencing technologies and pipelines, differences in assembly quality could impact SNP identification and tree topology. I recommend including a statement in the limitations section acknowledging this potential source of variability.

Response: We thank the reviewer for this important observation. We agree that the publicly available NCBI genome assemblies used for SNP-based phylogenetic analysis were generated using different sequencing platforms, assembly pipelines, and quality control parameters, which may introduce variability in assembly quality and, consequently, influence SNP detection and phylogenetic tree topology.

In response, we have added a statement in the Limitations section (Line: 355-357) acknowledging this as a potential source of technical variability. We also note that, despite this inherent limitation, the large number of genomes analyzed provides valuable insight into the broader phylogenetic structure and lineage distribution of *S. sonnei* and *S. flexneri*.

#4.**Comment:** The manuscript now includes a detailed description of the bioinformatics pipeline and key parameters for SNP calling, recombination filtering, and genome selection. To further enhance reproducibility, I recommend providing the actual step-by-step commands or scripts, either as a supplementary file or in a public repository (e.g., GitHub), for the WGS and phylogenetic analysis steps. This would allow other researchers to replicate the analysis and is considered best practice for large-scale genomic studies.

Response: We thank the reviewer for this valuable suggestion and fully agree that providing the exact step-by-step commands used for the WGS and phylogenetic analyses would further strengthen the reproducibility of the study.

In response, we will provide a supplementary file S1 (Line: 490-492) containing the detailed command-line workflow and key scripts used for genome processing, SNP

calling, recombination filtering, genome selection, and phylogenetic reconstruction. This supplementary material will include the principal software commands and relevant parameters to facilitate replication of the analysis by other researchers.

We agree that this represents best practice for large-scale genomic studies and appreciate the reviewer's recommendation to improve the transparency and reproducibility of our bioinformatics workflow.

#5.**Comment:** Please provide high-quality figures. For example, the text in Figure 3 is difficult to read and would benefit from improved resolution or larger fonts.

Response: We thank the reviewer for this helpful comment. Figure 3 has been revised using a higher-resolution image with enlarged text and labels to improve readability. In addition, all figures were rechecked to ensure adequate resolution and consistent formatting in the revised manuscript.

Re: Spectrum02824-25R2 (Escalation of CTX-M-Producing Extensively Drug-Resistant Shigella spp. in Kolkata, India, Following the COVID-19 Pandemic)

Dear Dr. Asish K Mukhopadhyay:

Your manuscript has been accepted, and I am forwarding it to the ASM production staff for publication. Your paper will first be checked to make sure all elements meet the technical requirements. ASM staff will contact you if anything needs to be revised before copyediting and production can begin. Otherwise, you will be notified when your proofs are ready to be viewed.

Data Availability: ASM policy requires that data be available to the public upon online posting of the article, so please verify all links to sequence records, if present, and make sure that each number retrieves the full record of the data. If a new accession number is not linked or a link is broken, provide production staff with the correct URL for the record. If the accession numbers for new data are not publicly accessible before the expected online posting of the article, publication may be delayed; please contact ASM production staff immediately with the expected release date.

Publication Fees: For information on publication fees and which article types have charges, please visit our website. We have partnered with Copyright Clearance Center (CCC) to collect author charges. If fees apply to your paper, you will receive a message from no-reply@copyright.com with further instructions. For questions related to paying charges through RightsLink, please contact CCC at ASM_Support@copyright.com or toll free at +1-877-622-5543. CCC makes every attempt to respond to all emails within 24 hours.

ASM Membership: Corresponding authors may join or renew ASM membership to obtain discounts on publication fees. Need to upgrade your membership level? Please contact Customer Service at Service@asmusa.org.

Embargo Policy: A press release may be issued as soon as the manuscript is posted on the Spectrum Latest Articles webpage. The corresponding author will receive an email with the subject line "ASM Journals Author Services Notification" when the article is available online.

The ASM Journals program strives for constant improvement in our submission and publication process. Please tell us how we can improve your experience by taking this quick Author Survey.

Thank you for submitting your paper to Spectrum.

Sincerely,
Reema Singh
Editor
Microbiology Spectrum
