# Supplementary material for: Escalation of CTX-M-producing extensively drug-resistant Shigella spp. in Kolkata, India, following the COVID-19 pandemic
Source: Microbiol Spectr. 2026 Jul 24;14(9):e02824-25. doi: 10.1128/spectrum.02824-25 (PMC13532320; doi:10.1128/spectrum.02824-25)
Supplement: Supplementary file 2 [file spectrum.02824-25-s0002.pdf]

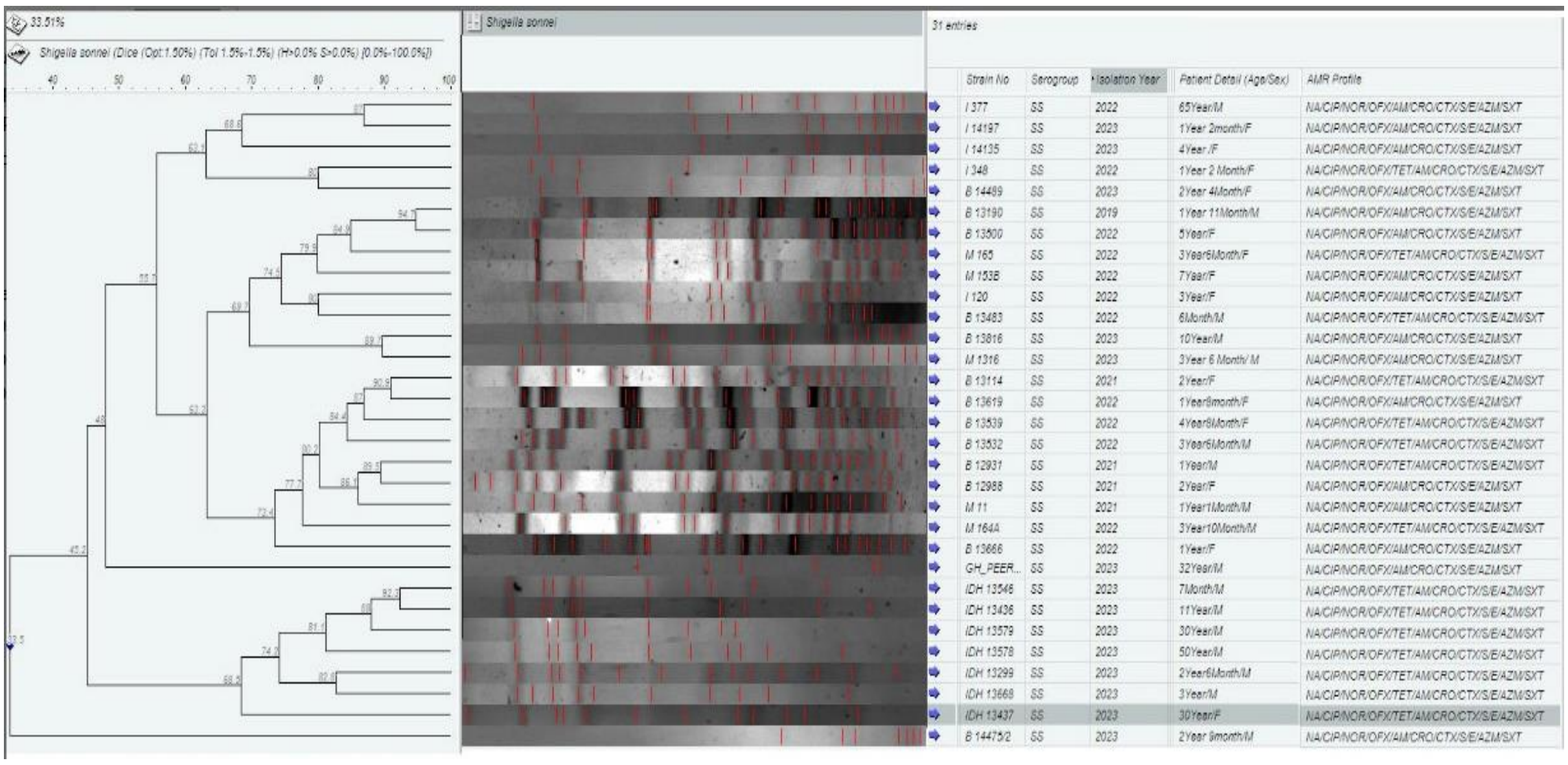

Fig. S1. PFGE-based clonal analysis of representative XDR *S. sonnei* XDR strains

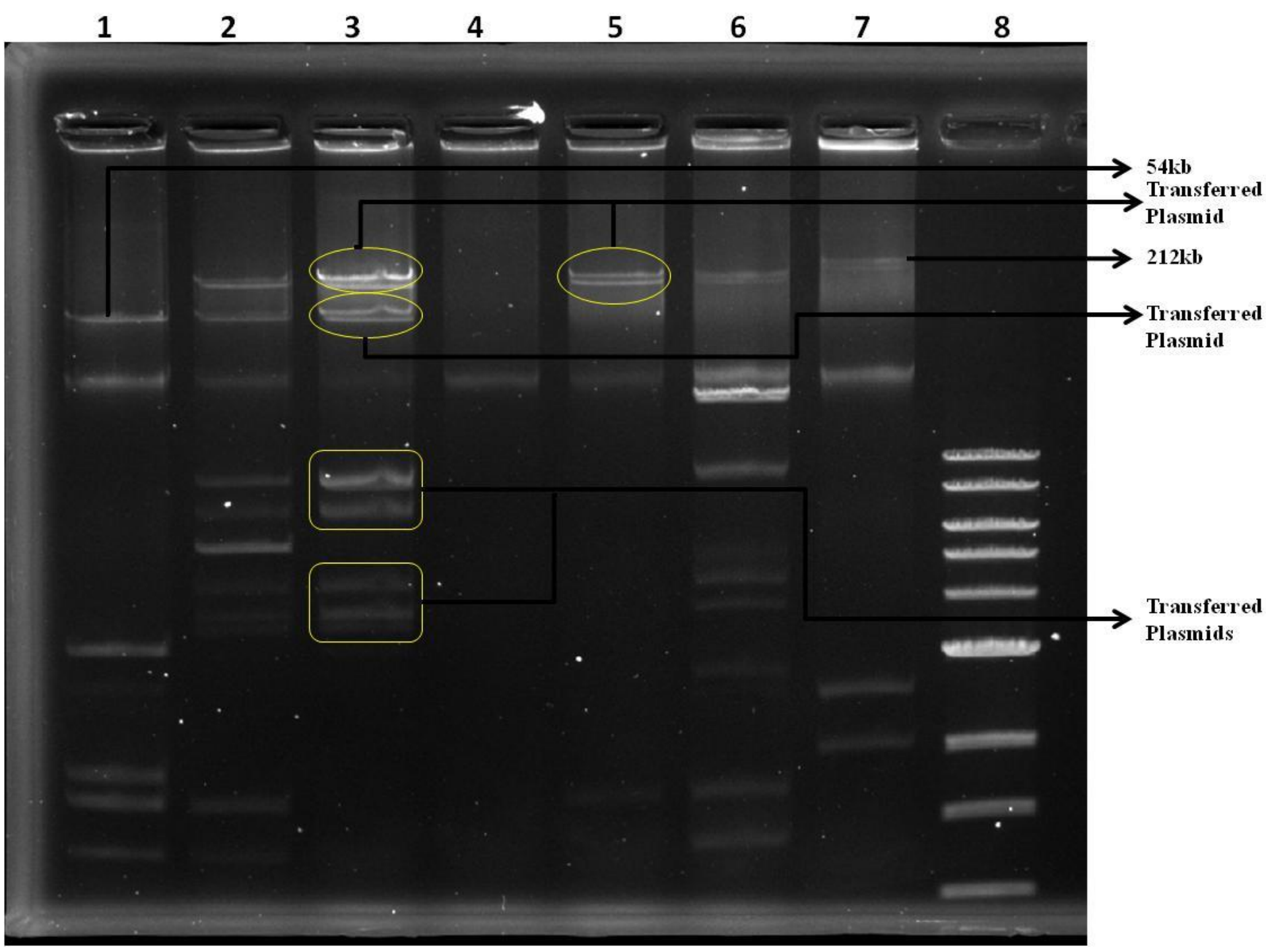

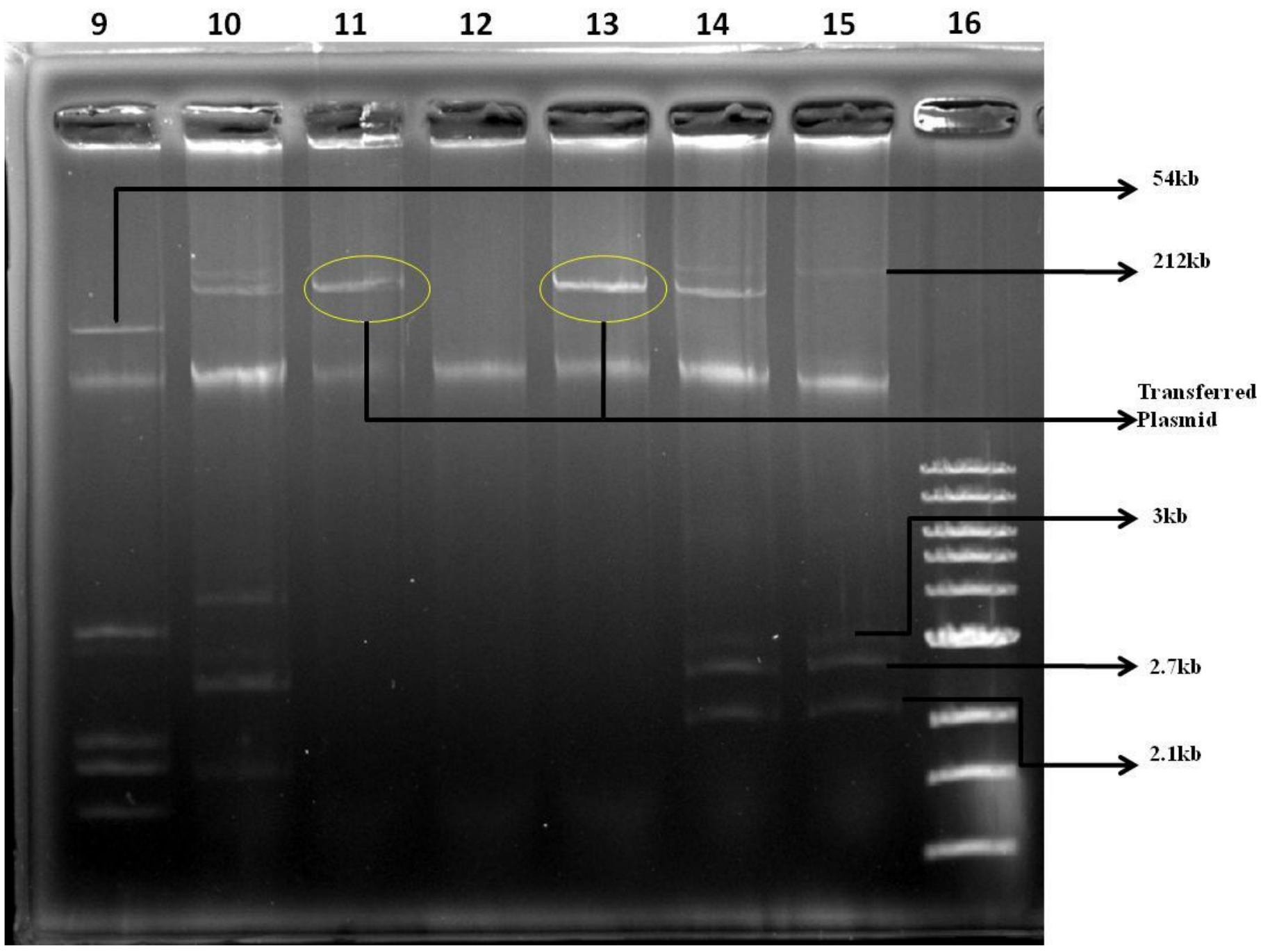

Fig. S2. plasmid profile of transconjugant and donor strains**.** Lane1, 9: *E. coli* V517, Lane2: *S. sonnei* M153B, Lane3: transconjugant M153B*J53, Lane4, 12: *E. coli* J53, Lane5: Transconjugant M164A*J53, Lane6: *S. sonnei* M164A, Lane7, 15: *S. flexneri* YSH6000, Lane8, 16: 1Kb DNA ladder, Lane10: *S. dysenteriae* I 182, Lane11: transconjugant I 182*J53, Lane13: transconjugant B 13618*J53, Lane14: *S. flexneri* B 13618

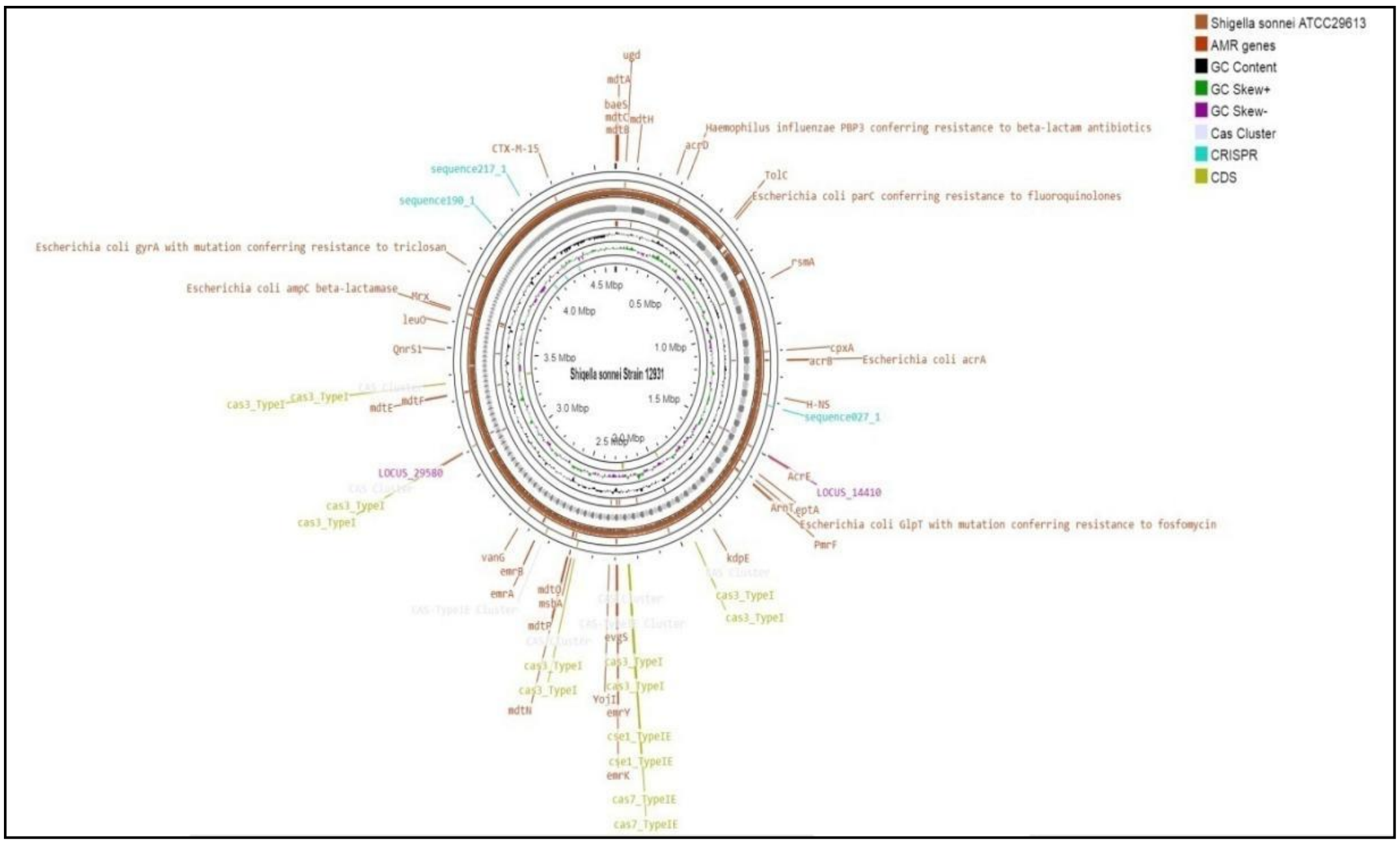

Fig. S3. Hybrid assembly showing that *bla*$_{CTXM-15}$ and *mphA* both were chromosome borne in *S. sonnei* (I 12931).

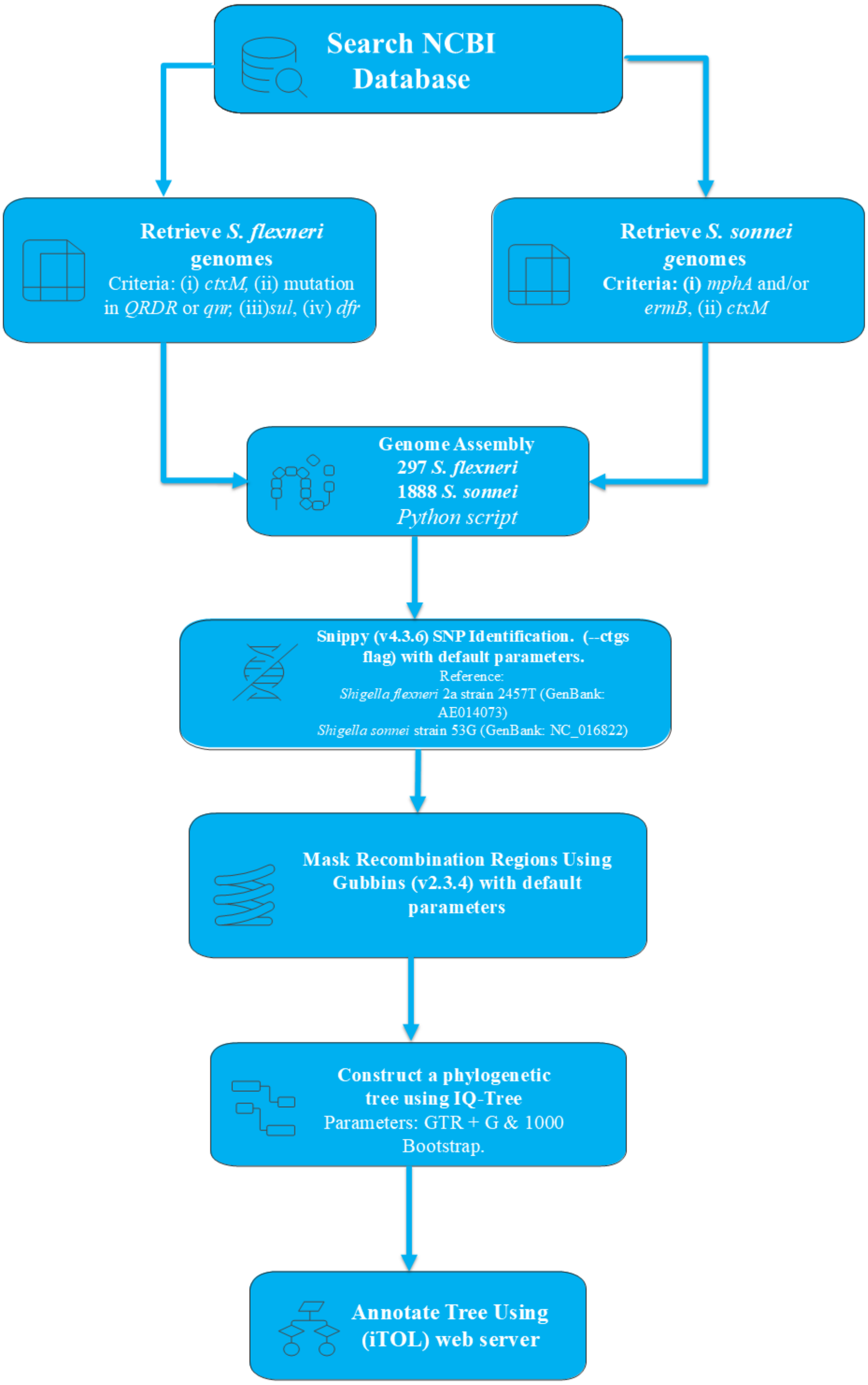

Fig. S4. A complete workflow diagram for phylogenetic analysis.
