# Supplementary material for: Escalation of CTX-M-producing extensively drug-resistant Shigella spp. in Kolkata, India, following the COVID-19 pandemic
Source: Microbiol Spectr. 2026 Jul 24;14(9):e02824-25. doi: 10.1128/spectrum.02824-25 (PMC13532320; doi:10.1128/spectrum.02824-25)
Supplement: Supplementary file 3 [file spectrum.02824-25-s0001.docx]

**Supplementary File S1.**

**Whole genome sequence (WGS) analysis-Step-by-step command workflow for hybrid genome assembly, polishing, QC, and annotation:**

**Step 1. Read assembly using Unicycler**

Both short Illumina paired-end reads and long Oxford Nanopore reads were used for de novo hybrid assembly using Unicycler.

Executable assembly command

unicycler -1 reads_R1.fastq.gz -2 reads_R2.fastq.gz -l long_reads.fastq.gz -o unicycler_output -t 8

**Step 2. Assembly quality evaluation using QUAST v5.0.2**

Assembly quality metrics including coverage, total length, N50, GC content, and number of contigs >500 bp were calculated using QUAST.

Executable QUAST command

quast assembly.fasta -r reference.fasta -o quast_output -t 8

Quality filtering criteria

Assemblies were retained if they met: Coverage ≥30×, N50 >20 kb, Contigs <300

**Step 3. Read mapping for Pilon polishing**

Illumina short reads were mapped back to the hybrid assembly. Pilon was used to correct base mismatches, small indels, and local misassemblies.

Executable Pilon command

pilon --genome assembly.fasta --frags aln.sorted.bam --output pilon_output --threads 8 --fix all

**Step 4. Genome annotation using RAST**

The polished FASTA assembly was uploaded to the RAST server.

RAST web workflow

Open <http://rast.nmpdr.org>, upload the file, select organism: *Shigella* spp., select annotation pipeline: RASTtk, download GenBank output (.gbk)

**Phylogenetic analysis-Step-by-step command workflow:**

<https://github.com/Pratanukayet/snp_phylogenetic_tree/tree/main>

**References:**

1. Wick RR, Judd LM, Gorrie CL, Holt KE. 2017. Unicycler: resolving bacterial genome assemblies from short and long sequencing reads. *PLoS Comput Biol* 13(6). <https://doi.org/10.1371/journal.pcbi.1005595>
2. Gurevich A, Saveliev V, Vyahhi N, Tesler G. 2013. QUAST: quality assessment tool for genome assemblies. *Bioinformatics* 29(8):1072–1075. <https://doi.org/10.1093/bioinformatics/btt086>
3. Walker BJ, Abeel T, Shea T, Priest M, Abouelliel A, Sakthikumar S, Cuomo CA, Zeng Q, Wortman J, Young SK, Earl AM. 2014. Pilon: an integrated tool for comprehensive microbial variant detection and genome assembly improvement. *PLoS One* 9(11). <https://doi.org/10.1371/journal.pone.0112963>
4. Aziz RK, Bartels D, Best AA, DeJongh M, Disz T, Edwards RA, Formsma K, Gerdes S, Glass EM, Kubal M, Meyer F, Olsen GJ, Olson R, Osterman AL, Overbeek RA, McNeil LK, Paarmann D, Paczian T, Parrello B, Pusch GD, Reich C, Stevens R, Vassieva O, Vonstein V, Wilke A, Zagnitko O. 2008. The RAST Server: rapid annotations using subsystems technology. *BMC Genomics* 9:75. <https://doi.org/10.1186/1471-2164-9-75>
